# The Discovery of Twenty-Eight New Encapsulin Sequences, Including Three in Anammox Bacteria

**DOI:** 10.1038/s41598-019-56533-5

**Published:** 2019-12-27

**Authors:** John C. Tracey, Maricela Coronado, Tobias W. Giessen, Maggie C. Y. Lau, Pamela A. Silver, Bess B. Ward

**Affiliations:** 1 Princeton University, Department of Geosciences, Guyot Hall, Princeton, NJ 08544 USA; 2 Department of Biomedical Engineering, University of Michigan, Ann Arbor, MI 48109 USA; 3 Harvard Medical School, Department of Systems Biology, Boston, MA 02115 USA; 4 Wyss Institute for Biologically Inspired Engineering, 3 Blackfan Circle, Boston, MA 02115 USA; 5 Institute of Deep-Sea Science and Engineering, Chinese Academy of Sciences, Sanya, Hainan China

**Keywords:** Phylogeny, Sequence annotation, Soil microbiology, Water microbiology

## Abstract

Many prokaryotes encode protein-based encapsulin nanocompartments, including anaerobic ammonium oxidizing (anammox) bacteria. This study expands the list of known anammox encapsulin systems from freshwater species to include the marine genus *Scalindua*. Two novel systems, identified in “*Candidatus Scalindua rubra*” and “*Candidatus Scalindua* sp. SCAELEC01 167” possess different architectures than previously studied freshwater anammox encapsulins. Characterization of the *S*. *rubra* encapsulin confirms that it can self-assemble to form compartments when heterologously expressed in *Escherichia coli*. BLASTp and HMMER searches of additional genomes and metagenomes spanning a range of environments returned 26 additional novel encapsulins, including a freshwater anammox encapsulin identified in “*Candidatus Brocadia caroliniensis*”. Phylogenetic analysis comparing these 28 new encapsulin sequences and cargo to that of their closest known relatives shows that encapsulins cluster by cargo protein type and therefore likely evolved together. Lastly, prokaryotic encapsulins may be more common and diverse than previously thought. Through searching a small sample size of all public metagenomes and genomes, many new encapsulin systems were unearthed by this study. This suggests that many additional encapsulins likely remain to be discovered.

## Introduction

Biological metabolisms often require the sequestration of toxic byproducts and the use of spatial barriers between incompatible reactions. Eukaryotes use lipid-based membrane bound organelles such as peroxisomes and lysosomes to achieve these functions. Many prokaryotes possess proteinaceous compartments that could confer some of the same advantages as eukaryotic organelles. In these systems protein, rather than lipid, polymers form the boundary between the compartment and the cytosol. The proposed advantages of these compartments include the ability to concentrate enzymes and metabolites at one location, prevent damage from toxic metabolic intermediates or byproducts, and perform incompatible reactions simultaneously^1^.

Prokaryotes use many types of protein compartments, such as carboxysomes^2^, ferritins^3^, proteins analogous to eukaryotic cytoskeleton^4^, and small micro-compartments dubbed encapsulin nanocompartments^1,5^. Encapsulin nanocompartments are icosahedral hollow protein complexes that are currently known to occur in two forms, one composed of 60 encapsulin subunits with a diameter of 20–24 nm and another of 180 subunits with a diameter of 30–40 nm^1^. Each encapsulin nanocompartment typically encapsulates core cargo enzymes, which assemble with the encapsulin shell through binding sequences on their C terminus^5,6^, as well as occasional secondary cargo proteins. For most encapsulin systems, core and secondary cargo proteins can be differentiated based on three consensus sequences^1^. Prior to this work, 913 encapsulin systems were known from an extensive search of the NCBI non-redundant (nr) protein database^1^.

Four of the 913 encapsulins described by Giessen and Silver^1^ were found in anaerobic ammonium oxidizing (anammox) bacteria. The anammox reaction is thought to be a three-step reaction where the net reaction is the oxidation of ammonium with nitrite to create dinitrogen gas^7^. These three steps and the net reaction, listed in order with the enzymes involved, are:

$$\begin{array}{cc}{{{\rm{N}}{\rm{O}}}_{2}}^{-}+{2{\rm{H}}}^{+}+{{\rm{e}}}^{-}\leftrightharpoons {\rm{N}}{\rm{O}}+{{\rm{H}}}_{2}{\rm{O}} & ({\rm{N}}{\rm{i}}{\rm{t}}{\rm{r}}{\rm{i}}{\rm{t}}{\rm{e}}\,{\rm{r}}{\rm{e}}{\rm{d}}{\rm{u}}{\rm{c}}{\rm{t}}{\rm{a}}{\rm{s}}{\rm{e}},\,{\rm{N}}{\rm{i}}{\rm{R}}\,{\rm{o}}{\rm{r}}\,{\rm{N}}{\rm{i}}{\rm{t}}{\rm{r}}{\rm{i}}{\rm{t}}{\rm{e}}\,{\rm{r}}{\rm{e}}{\rm{d}}{\rm{u}}{\rm{c}}{\rm{t}}{\rm{a}}{\rm{s}}{\rm{e}}\,{\rm{a}}{\rm{n}}{\rm{a}}{\rm{l}}{\rm{o}}{\rm{g}})\\ {\rm{N}}{\rm{O}}+{{{\rm{N}}{\rm{H}}}_{4}}^{+}+{2{\rm{H}}}^{+}+{3{\rm{e}}}^{-}\leftrightharpoons {{\rm{N}}}_{2}{{\rm{H}}}_{4}\,{+{\rm{H}}}_{2}{\rm{O}} & ({\rm{H}}{\rm{y}}{\rm{d}}{\rm{r}}{\rm{a}}{\rm{z}}{\rm{i}}{\rm{n}}{\rm{e}}\,{\rm{s}}{\rm{y}}{\rm{n}}{\rm{t}}{\rm{h}}{\rm{a}}{\rm{s}}{\rm{e}},\,{\rm{H}}{\rm{Z}}{\rm{S}})\\ {{\rm{N}}}_{2}{{\rm{H}}}_{4}\leftrightharpoons {{\rm{N}}}_{2}+{4{\rm{H}}}^{+}+{4{\rm{e}}}^{-} & ({\rm{H}}{\rm{y}}{\rm{d}}{\rm{r}}{\rm{a}}{\rm{z}}{\rm{i}}{\rm{n}}{\rm{e}}\,{\rm{d}}{\rm{e}}{\rm{h}}{\rm{y}}{\rm{d}}{\rm{r}}{\rm{o}}{\rm{g}}{\rm{e}}{\rm{n}}{\rm{a}}{\rm{s}}{\rm{e}},\,{\rm{H}}{\rm{D}}{\rm{H}})\\ {{{\rm{N}}{\rm{O}}}_{2}}^{-}+{{{\rm{N}}{\rm{H}}}_{4}}^{+}\leftrightharpoons {{\rm{N}}}_{2}+{2{\rm{H}}}_{2}{\rm{O}} & ({\rm{N}}{\rm{e}}{\rm{t}}\,{\rm{r}}{\rm{e}}{\rm{a}}{\rm{c}}{\rm{t}}{\rm{i}}{\rm{o}}{\rm{n}})\end{array}$$

Hydrazine (N_2_H_4_) is a very reactive compound used as a fuel in the first rockets. Due to hydrazine’s high reactivity, it was hypothesized that the anammox reaction is performed inside a unique membrane-bound organelle called the anammoxosome, which occupies most of the cell volume of anammox bacteria^8,9^. This hypothesis has been indirectly confirmed through the immunogold localization of HZS and HDH to the anammoxosome^10^ as well as *in-vitro* evidence that isolated anammoxosomes can perform the anammox reaction^11^. The anammoxosome membrane is composed of unique ladderane lipids, whose wavy structure consisting of fused cyclobutane and cyclohexane rings allows these lipids to be densely packed in membranes. This membrane structure is thought to increase the anammoxosome membrane’s impermeability towards toxic intermediates and/or prevent the loss of the proton motive force across the anammoxosome membrane due to diffusion^9,12,13^.

All known anammox bacteria reside in a deeply branching monophyletic clade in the phylum Planctomycetes^13^. Five anammox genera have been described to date from environments including wastewater treatment plants, lakes, marine sediments, and the three large marine oxygen minimum zones (OMZs) found in the Eastern Tropical North Pacific (ETNP), Eastern Tropical South Pacific (ETSP), and the Arabian Sea (AS). The genera “*Candidatus Kuenenia,*” “*Brocadia,*” “*Jettenia,*” and “*Anammoxoglobus*” have all been enriched from freshwater environments, mainly wastewater treatment plant outflows. The last genus, “*Candidatus Scalindua*,” predominates in marine sediments and OMZs^8^. While these five genera are all within the same family, “Candidatus Brocadiaceae,” the marine *Scalindua* species form a separate outgroup from the freshwater genera^14^.

**Figure 1:**
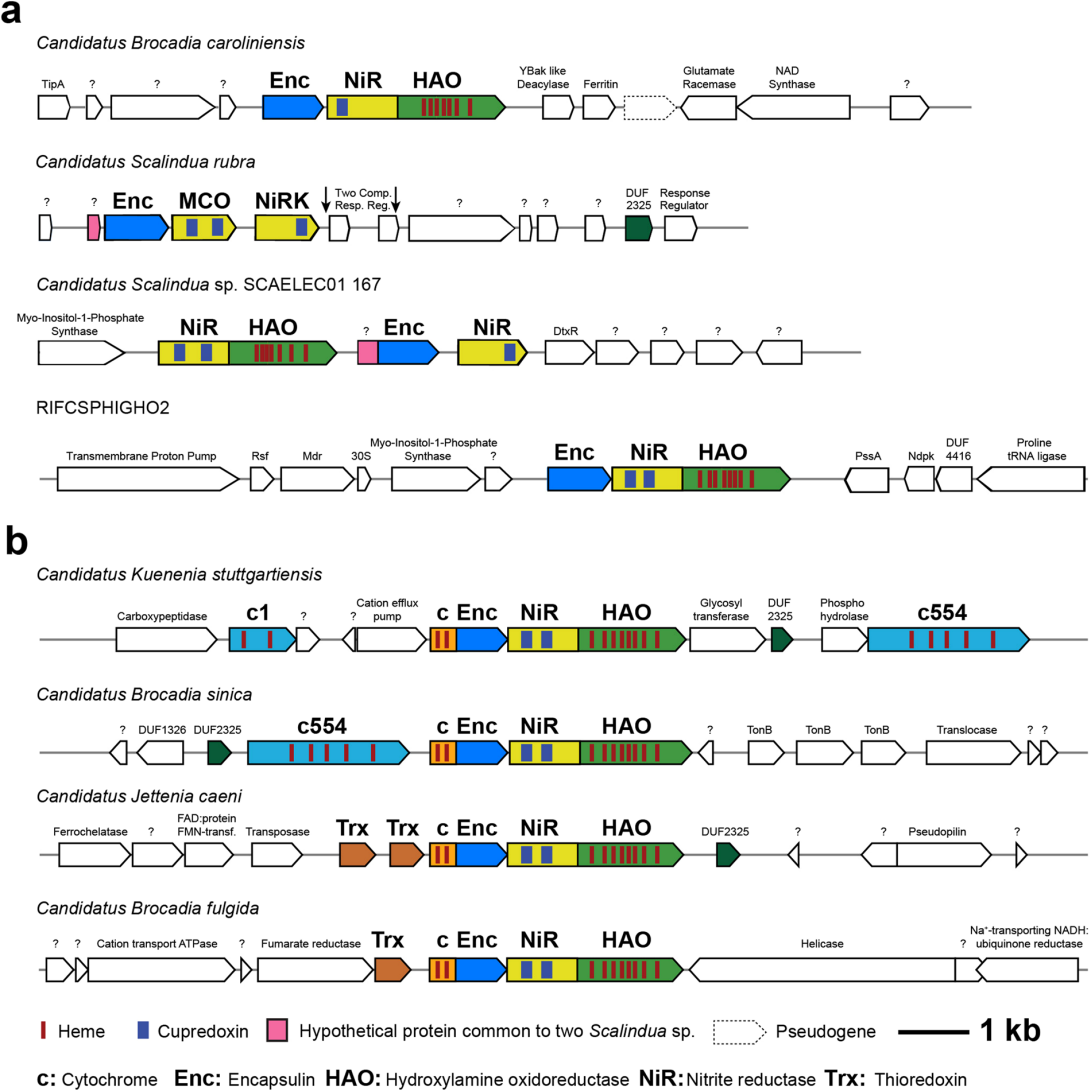
Comparison of newly discovered and previously known Planctomycete encapsulins. (**a**) Four newly discovered Planctomycete encapsulin systems. The *B*. *caroliniensis* and uncultured Planctomycete RIFCSPHIGHO2 systems contain an encapsulin followed by a nitrite reductase/hydroxylamine oxidoreductase (NiR/HAO) fusion protein. The *S*. *rubra* system consists of an encapsulin, a multi-copper oxidase (MCO), and a downstream NiRK. The *Scalindua* SCAELEC01 167 system consists of a NiR/HAO fusion protein, an encapsulin fused to a hypothetical protein also found immediately before the *S*. *rubra* encapsulin, and a nitrite reductase. (**b**) Previously known encapsulin systems discovered in freshwater anammox genera (figure reproduced with permission from ref. ^1^). These systems consist of a cytochrome c/encapsulin fusion protein (cEnc) followed by a NiR/HAO fusion protein. The blue bars represent cupredoxin copper binding domains and the red bars represent iron rich heme moieties. DUF2325, Trx, c1, and c554 are possible secondary cargo proteins.

Four freshwater anammox species possess an encapsulin system with an unusual double fusion protein architecture^1^. These freshwater anammox species contain an encapsulin/cytochrome c fusion protein (cEnc) encoded immediately before a nitrite reductase-like (NiR)/Hydroxylamine Oxidoreductase (HAO)*-*like fusion cargo protein (Fig. 1b). The presence of a NiR/HAO fusion protein hints at a potential role for the encapsulin system in the anammox reaction through the nitrite reductase. Anammox bacteria contain many HAO-like proteins; for example, the freshwater species *K*. *stuttgartiensis* encodes ten^15^. The functions of these proteins in *K*. *stuttgartiensis* vary widely; HDH is a HAO-like protein specifically tuned to oxidize hydrazine to N_2_ gas^16^, whereas another *Kuenenia* HAO-like protein generates NO from hydroxylamine (NH_2_OH)^17^. It is hypothesized, based on experimental evidence from nitrifying organisms, that the presence of a C terminal tyrosine indicates whether a HAO-like protein is tuned for oxidative or reductive catalysis^18–23^. The *K*. *stuttgartiensis* encapsulin associated fusion cargo HAO lacks a C terminal tyrosine^22^, indicating that its HAO-like domain is likely involved in the reduction of nitrogenous compounds.

In addition to the phylum Planctomycetes, encapsulins are found in 14 bacterial and two archaeal phyla from species across a wide range of environments^1^. In order to explore additional encapsulin diversity, we searched for new encapsulins in environmental metagenomes outside the scope of previous searches. These searches were performed using the HMMER algorithm to take advantage of HMMER’s unique ability to search metagenomic data with a profile of all 913 known encapsulins^1^ with greater sensitivity to remote homologs and comparable speed to BLAST^24^.

## Methods

### BLASTp Searches for Planctomycete Encapsulins

Each of the putative four freshwater anammox encapsulin and fusion cargo amino acid sequences was used as a query for a BLASTp search against the NCBI non-redundant (nr), environmental metagenome (env_nr), and patented (pat) protein sequence databases using default parameters. The percent similarities of the new anammox encapsulins and cargo proteins to previously known freshwater anammox cEnc and NiR/HAO fusion proteins were determined using Clustal Omega with the default parameters^25^. Copper binding cupredoxin domains and iron rich heme binding sites containing CxxCH motifs were located via Conserved Domain (CD) search of the CDD v3.16 database using the default settings^26^. The encapsulin core cargo and two secondary cargo consensus sequences^1^ were used as queries for BLASTp searches to discover new anammox encapsulin cargo proteins. These sequences are DGSLGTIGSLKGE (core cargo sequence), RGSVVPAGGAAV (FolB secondary cargo sequence), and DGAPPAAGGAL (BfrB secondary cargo sequence). BLASTp parameters for the cargo consensus sequences searches were as follows: the PAM30 matrix was used with the word size set to 2, the cost to open a gap set to 9, and the expect threshold equal to 200,000.

### Characterization of the *Scalindua rubra* Encapsulin

**Figure 2:**
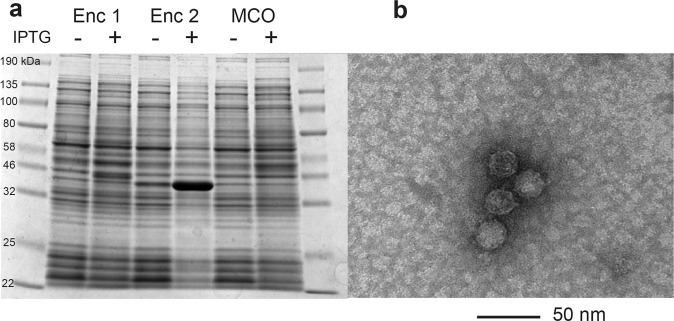
The *S*. *rubra* encapsulin monomer can self-assemble into a spherical polymer 25 nm in diameter. (**a**) Protein gel electrophoresis reveals that a protein with the predicted molecular mass (34 kDa) of the *S*. *rubra* encapsulin can be expressed in and isolated from *E*. *coli* lysate. Enc 1 refers to a protein beginning at the first of two possible translation start sites, Enc 2 refers to a protein that begins at the second site. (**b**) TEM imaging of a solution containing purified *S*. *rubra* encapsulin reveals that the *S*. *rubra* encapsulin monomer self-assembles into a spherical polymer with a diameter of 25 nm.

The *Scalindua rubra* encapsulin system was cloned using Gibson Assembly. gBlock gene fragments (IDT Inc., Iowa, USA) were created that (1) encode the *E*. *coli* codon optimized encapsulin gene and multi-copper oxidase cargo (*mco*) together so that they would be under one promoter, (2) contain only the *S*. *rubra* encapsulin, or (3) contain only the *S*. *rubra mco* with a C terminal His tag. Each gBlock contains a 25 bp overlap for direct assembly. The gBlocks were combined with a NdeI and PacI digested pETDuet1 plasmid to yield assembled expression constructs. Electrocompetent *E*. *coli* DH10B cells (Invitrogen) were transformed with the expression constructs and confirmed by sequencing (Genewiz). Assembled and sequence-confirmed plasmids (0.5 ng total DNA) (see Supplementary Materials for sequencing files) were then used to transform chemically competent *E*. *coli* BL21 (DE3) cells (Invitrogen). Expression experiments were carried out in LB broth supplemented with ampicillin (100 mg mL^−1^). For protein expressions 50 mL LB was inoculated (1:50) using an over-night culture, grown at 37 °C and 200 rpm to an OD_600_ of 0.5, then induced with IPTG (final concentration: 0.1 mM). Cultures were grown at 30 °C for 18 h, harvested via centrifugation (4,000 rpm, 15 min, 4 °C) and the pellets either immediately used or flash-frozen in liquid nitrogen and stored at −20 °C. For encapsulin protein purifications, pellets were resuspended in 5 mL PBS (pH 7.4) followed by the addition of lysozyme (1 mg mL^−1^ final concentration) and DNase I (1 μg mL^−1^ final concentration). This solution was incubated for 20 min on ice, then sonicated using a 550 Sonic Dismembrator (Fisher Scientific) using power level 3.5, pulse time of 8 s, an interval of 10 s, and total pulse time of 3 min. Cell debris was removed through centrifugation (8,000 rpm, 15 min, 4 °C). To test induction, a fraction of this lysate was run on a Novex Tris-Glycine gel following standard protocols, stained with Coomassie Blue and compared with lysates resulting from uninduced cultures (Figs. 2a and S1–3). A 5 mL fraction of the encapsulin (derived from the separate promoter gBlocks) lysate was prepared for TEM analysis by PEG precipitation using the sequential addition of 0.15 g NaCl and 0.4 g PEG-8000 (8% w/v final concentration) followed by incubation on ice for 30 min. Precipitate was collected through centrifugation (8,000 rpm, 15 min, 4 °C), resuspended in PBS buffer, filtered using a 0.2 μm syringe filter (Fisher Scientific) and then centrifuged (8,000 rpm, 15 min, 4 °C). The pellet was resuspended in PBS buffer and subjected to size exclusion chromatography performed with an AKTA Explorer 10 (GE Healthcare Life Sciences) equipped with a HiPrep 16/60 Sephacryl S-500 HR column (GE Healthcare Life Sciences) using a flow rate of 1 mL min^−1^. Fractions were evaluated using SDS–PAGE (Figs. S4 and 5) and encapsulin containing fractions were combined and concentrated using Amicon 100,000 NMWL filters. Samples were stained for TEM using uranyl formate and visualized on an FEI Tecnai G2 at 80 kV (Figs. 2b and S6).

### HMMER Searches for *Scalindua* and Other Novel Encapsulins in Environmental Metagenomes

Multiple amino acid and DNA sequence alignments containing 913 putative encapsulins^1^ and the *S*. *rubra* encapsulin were created using CLUSTAL-OMEGA^27^. These alignments were then converted into profile HMMs using HMMER v3.1 b2^24^. Research collaborators generously provided three metagenomic datasets to search for a *Scalindua* encapsulin and other novel encapsulins: (1) particulate material from OMZ seawater (ETSP) at 40, 80, 200 & 300 m depths provided by Xin Sun^28^, (2) an anammox enrichment from a Swedish fjord^29^, and (3) a Dominican saltwater aquifer where *Scalindua* hits had been observed by Jenn Macalady and Zena Cardman (Table S1). Twenty-five additional metagenomic datasets (Table S1), including coastal waters, estuaries, and bays (n = 13), soil and root microbiome samples (n = 5), OMZs (n = 3), the open ocean (n = 2), and the human microbiome (n = 2) were downloaded from MG-RAST^30^ and NCBI’s Sequence Read Archive to search for novel encapsulins in a wide variety of environments. Each metagenome’s DNA and amino acid sequences were searched for encapsulins by performing a HMMER search with the 914 encapsulin HMM profile as query. The default parameters were used for all HMMER searches and E-values less than 10^−5^ were considered significant. To further validate positive HMMER hits, all metagenomes with positive HMMER hits were assembled again with IDBA_UD v1.1.1^31^ and open reading frames (ORFs) were predicted using Prodigal v2.6.3^32^. HMMER searches were performed on these files using the same parameters as above.

Encapsulins and HK97 viral capsid proteins, one of the major classifications of capsid monomers^33,34^, are largely identical in appearance, yet only have weak similarity in amino acid sequence^5,35^. Nevertheless, in order to definitively verify that the HMMER hits were encapsulins and not viral capsid proteins, each gene within 10 kb of a potential encapsulin was used as a query for BLASTp searches with the default parameters against the nr database. If the best matching BLASTp hit to any gene within 10 kb was viral in origin (according to the annotation in the NCBI database) the potential encapsulin sequence present on that contig was discarded as a viral homolog. Many contigs did not extend for 10 kb on each side of the possible encapsulin. In these cases, all genes on the contig were searched. In order to double check that the HMMER hits had homology to encapsulins, each significant non-viral HMMER hit was used as a query for a NCBI CD-search with the default parameters against the CDD v3.16 database. Since all encapsulins are part of the Linocin M18 family (protein family PF04454), homology to this family with an E-value less than 10^−5^ was considered significant.

In order to identify the HMMER hits, each HMMER hit as well as the full-length protein sequence, from the metagenome, that contained each hit was used as a query for a BLASTp search against the nr database. Due to the voluminous hits that these searches returned, only the top three best matching protein sequences for each of the two searches are reported here. In order to check if these sequences were non-viral, the assemblies for each of the top three BLASTp hits for each search were examined. If any gene within 10 kb of the match was annotated as a viral protein, that match was not reported as a new encapsulin.

In addition to discovering new encapsulins through reviewing BLASTp results with HMMER hits as queries, this study also sought to determine if the metagenome derived full-length encapsulin sequences themselves were new encapsulins. This determination depends on (1) how similar the full-length sequences are to each other and (2) how similar the full-length sequences are to the best matched BLASTp sequence. Question (1) was addressed through creating pairwise alignments using Jalview with default parameters.

Question (2) was addressed by pairwise comparison of full-length sequences to the best BLASTp result. The typical percent identity thresholds for taxonomic classifications are defined for 16S rRNA genes as >98.65% to be members of the same species and >95% to be within the same genus^36^. Since this study involves protein sequences, an alternative threshold was found in the previously calculated percent similarities between freshwater anammox encapsulins. If the percent identity between two full-length sequences (question 1) or a full-length sequence and a BLASTp result (question 2) was 89% or lower (the percent identity between the *Brocadia caroliniensis* and *B*. *fulgida* encapsulins and the highest encapsulin percent similarity between two distinct species) then those two full-length encapsulin sequences were considered to be from separate species.

### Construction of the Encapsulin Phylogenetic Tree

All previously known encapsulins^1^, the four new anammox and Planctomycete encapsulins, the full-length encapsulin sequences containing the HMMER hits, and the new encapsulins found by BLASTp were aligned using MAAFT^25^ with the default parameters for protein sequences. The alignment was trimmed using TrimAl v1.3^37^ in Phylemon v2.0^38^ with the gap threshold set at 0.05. The phylogenetic tree was created using Fasttree v2.1.10^39^ with the WAG model of protein evolution. Lengths were rescaled to optimize the Gamma20 likelihood after using the CAT approximation to optimize the tree. Before running Fasttree, Prottest v3.4.2^40^ was used to select the most appropriate model of evolution among Fasttree’s three options – the WAG, JTT, and LG models. The resulting tree was visualized, annotated, and pruned to include only those sequences closely related to the newly discovered encapsulins in ITOL v4^41^. Cargo proteins were identified by examining the assemblies that contain each encapsulin. The gene encoded immediately before the encapsulin, except in the case of the anammox species, was classified by cargo type according to Giessen and Silver^1^.

## Results

### BLASTp Searches for Planctomycete Encapsulins

BLASTp searches of the nr database with the previously known freshwater anammox encapsulin and cargo sequences as queries returned two potential *Scalindua* encapsulin systems whose structures hint at a role in the anammox process (Fig. 1a). The first putative encapsulin was previously annotated as a hypothetical protein from “*Candidatus S*. *rubra*,” a proposed species found in a metagenome from the interface of a hypersaline brine pool in the Red Sea^42^. The *S*. *rubra* encapsulin is 306 amino acids long and is 49–52% identical to previously identified freshwater encapsulins at the amino acid level (Fig. S7a). Unlike the freshwater anammox encapsulins, the *S*. *rubra* encapsulin is not fused to a cytochrome domain. The encapsulin contains two possible transcription start sites, a GTG start codon located at the beginning of the DNA sequence, and an ATG located 63 residues downstream (boldface in S1).

Encoded 17 bp downstream from the putative encapsulin is a multi-copper oxidase (MCO) that is between 54–59% identical to the NiR portion of the freshwater fusion cargo proteins. CD-searches reported that the *mco* gene aligns to the cl19115, cupredoxin superfamily, which contains type I copper centers that are typically involved in inter-molecular electron transfer reactions, as well as the COG2132 SufI family of multi-copper oxidases. Although not explicitly annotated as a nitrite reductase, this gene might be a functional nitrite reductase because one of the two cupredoxin domains (amino acids 65–190 out of 324) was also described by CD-search as a NiR*-*like laccase. Positioned 280 bp further downstream is a NiRK nitrite reductase, which is 22–23% similar to the nitrite reductase found in the freshwater anammox fusion protein (Fig. S7b). BLASTp searches with the FolB secondary cargo consensus sequence as query returned that this downstream NiRK contains a VPAGGAAV sequence or two-thirds of the FolB consensus sequence. This sequence, like almost all other secondary cargo sequences^1^ is located at the C terminal of the protein spanning amino acids 289–296 out of 344. Canonically, two cupredoxin domains are required for a functional nitrite reductase. When the NiRK was examined by CD-search, the results returned a specific hit to only cupredoxin domain two of a nitrite reductase, while a hit to cupredoxin domain one was obtained among the non-specific matches. As a result, the annotation of this gene as NiRK should be interpreted as provisional.

Due to the NiRK’s distance from the encapsulin, the most likely core cargo protein in *S*. *rubra* is the multicopper oxidase. Although the MCO protein does not contain the core cargo consensus sequence, it is not unusual for anammox core cargo proteins to lack this sequence^1^.

The NiRK, as evidenced by the presence of the FolB sequence, could function as a secondary cargo protein. The lack of a HAO-like cargo protein was confirmed by BLASTp searches with previously known freshwater anammox cargo sequences as queries. These searches returned seven *Scalindua* HAO proteins (Table S2); however, these *Scalindua* genes are either not from *Scalindua rubra* or are encoded on different contigs than the one containing an encapsulin. As a result, no conclusive evidence of a HAO*-*like cargo was found near the putative *S*. *rubra* encapsulin.

Another *Scalindua* encapsulin was discovered in “*Candidatus Scalindua* sp. SCAELEC01 167,” a proposed species from a lab-scale microbial electrolysis cell metagenome^43^. This encapsulin system fuses elements of all other anammox encapsulin systems. For example, the *S*. SCAELEC01 167 system contains both a NiR/HAO fusion cargo protein and a separate NiR cargo protein that is 73% identical to the *S*. *rubra* NiRK (Figs. 1a and S7b). Like the *S*. *rubra* NiRK, the *S*. SCAELEC01 167 NiR contains only a specific hit to cupredoxin domain two of a nitrite reductase when examined with CD-search. Similarly, the *S*. SCAELEC01 167 system contains part of the FolB secondary cargo sequence at the C terminal, in this case VPAGCAAV instead of the canonical sequence (VPAGGAAV) possessed by the *S*. *rubra* NiRK. *S*. SCAELEC01, like the RIFCSPHIGHO2_02_Full_50_42 system, also encodes a myo-inositol-1-phosphate synthase immediately before the fusion NiR/HAO cargo protein. This enzyme catalyzes the creation of the widespread signaling molecule inositol-1-phosphate from glucose-6-phosphate. Due to its proximity, the SCAELEC01 encapsulin system may be regulated by inositol-1-phosphate.

The SCAELEC01 167 encapsulin itself is a fusion protein between a hypothetical protein and the encapsulin monomer. When the sequence of this hypothetical protein was searched against the nr database, its best matching sequence was a hypothetical protein encoded immediately before the *Scalindua rubra* encapsulin. While the function of this protein is unknown, its proximity to the *Scalindua* encapsulins implies that it may play an important role in encapsulin function.

The same BLASTp searches that revealed the *Scalindua* encapsulins discovered a freshwater encapsulin system in a “*Candidatus Brocadia caroliniensis*” genome recovered from a wastewater treatment plant in New York City^44^. As in the *Scalindua* systems, the *B*. *caroliniensis* encapsulin is not fused to a cytochrome domain; however, the *B*. *caroliniensis* system does contain a NiR/HAO fusion protein (Fig. 1a). The *B*. *caroliniensis* encapsulin is 71–89% similar to the other freshwater anammox encapsulins (Fig. S7a). The *B*. *caroliniensis* fusion cargo protein is 70–85% similar to the freshwater cargo proteins (Fig. S7b).

A novel Planctomycete encapsulin system was also discovered in Planctomycetes bacterium RIFCSPHIGHO2_02_Full_50_42 (RIF), which was detected in a shallow, anoxic aquifer adjacent to the Colorado River^45^. This system, like the *S*. *rubra*, *S*. SCAELEC01, and *B*. *caroliniensis* systems, does not contain a cEnc fusion protein. However, like the freshwater anammox encapsulins, the RIF system does include a NiR/HAO fusion protein (Fig. 1a). BLASTp searches with the core cargo consensus sequence as query returned that the fusion NiR/HAO contains a TIGS sequence or 31% of the core cargo consensus sequence located at the C terminus, spanning amino acids 838–841 out of 867. The RIF encapsulin is 64–71% identical to both the freshwater and *Scalindua* encapsulins (Fig. S7a). The RIF cargo protein is 62–68% identical to the freshwater fusion cargo proteins and the *S*. *rubra* MCO (Fig. S7b). Like *S*. SCAELEC01, the RIF encapsulin also is encoded near a myo-inositol-1-phosphate synthase. The architecture of this encapsulin system, an encapsulin and fusion NiR/HAO, suggests that this uncultured RIF Planctomycete could be an anammox bacterium.

### Characterization of the *Scalindua rubra* Encapsulin

The putative *Scalindua rubra* encapsulin system was heterologously expressed in *E*. *coli* to examine if the monomer and cargo could assemble into a nanocompartment. Unfortunately, no expression of either the encapsulin or *mco* was observed when each gene was expressed under the same promoter (See Figs. S1 and 2). However, protein gel electrophoresis showed that a protein with the expected molecular mass of the *S*. *rubra* encapsulin (34 kDa as predicted by ExPASy)^46^ was present in the crude lysate when the *S*. *rubra* encapsulin and *mco* were under the control of separate promoters. Of the two possible translation start sites within the encapsulin, the second shorter site responded strongly to IPTG addition. The first site and the MCO (36 kDa predicted molecular mass) were not expressed at levels above the background bands (Figs. 2a and S3). TEM analysis of purified lysate revealed spherical particles approximately 25 nm in diameter, confirming that the *Scalindua rubra* putative encapsulin monomer can self-assemble to form protein nanocompartments (Fig. 2b).

### HMMER Searches for *Scalindua* and Other Novel Encapsulins in Environmental Metagenomes

**Figure 3:**
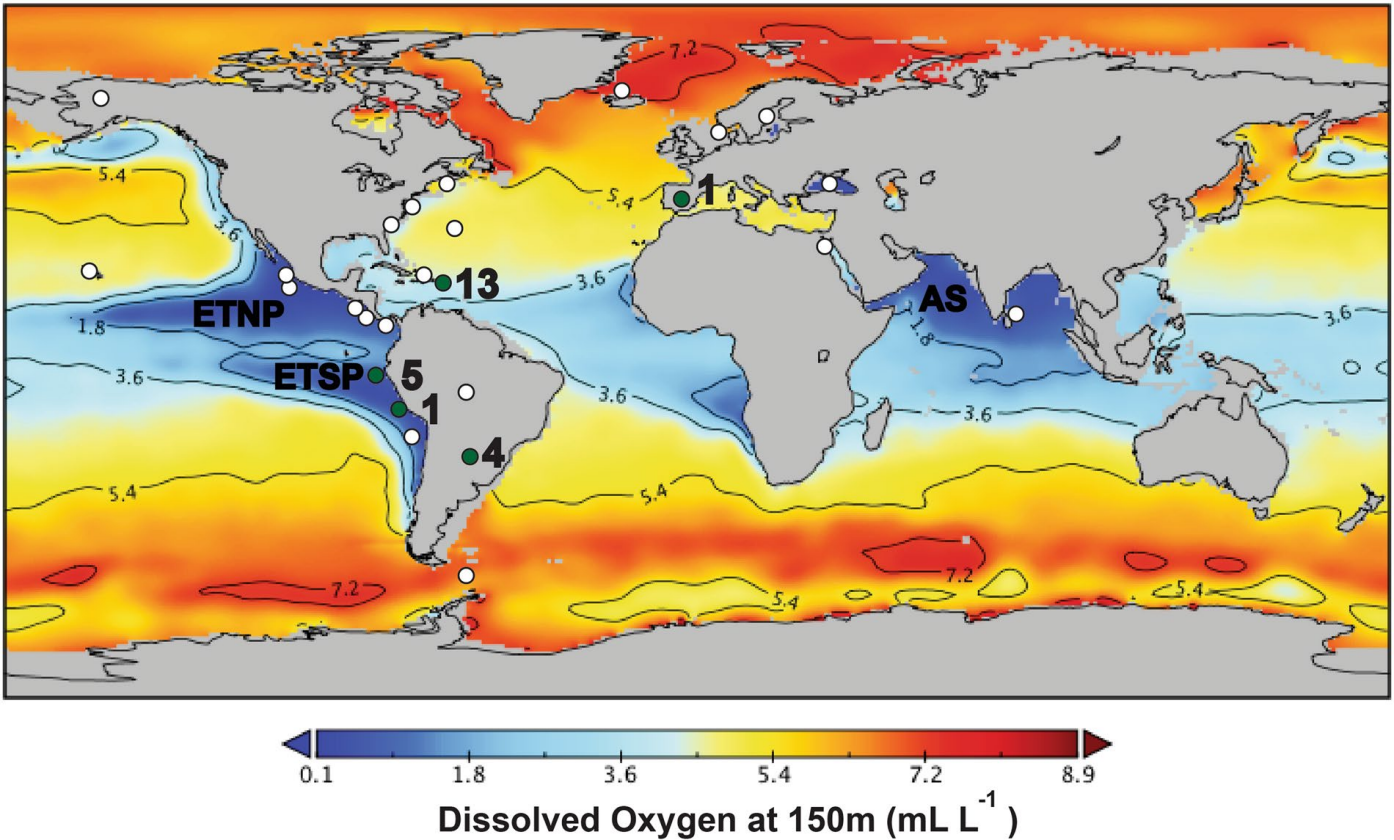
HMMER searches of OMZ metagenomes, as well as other environmental metagenomes, with an HMM profile of all 913 previously known encapsulins and the *Scalindua rubra* encapsulin as query returned twenty-four new encapsulins. Green points indicate locations where new encapsulins were discovered while white points indicate locations where no significant HMMER hits were observed. Black text indicates the number of novel non-viral encapsulins discovered at a location.

**Figure 4:**
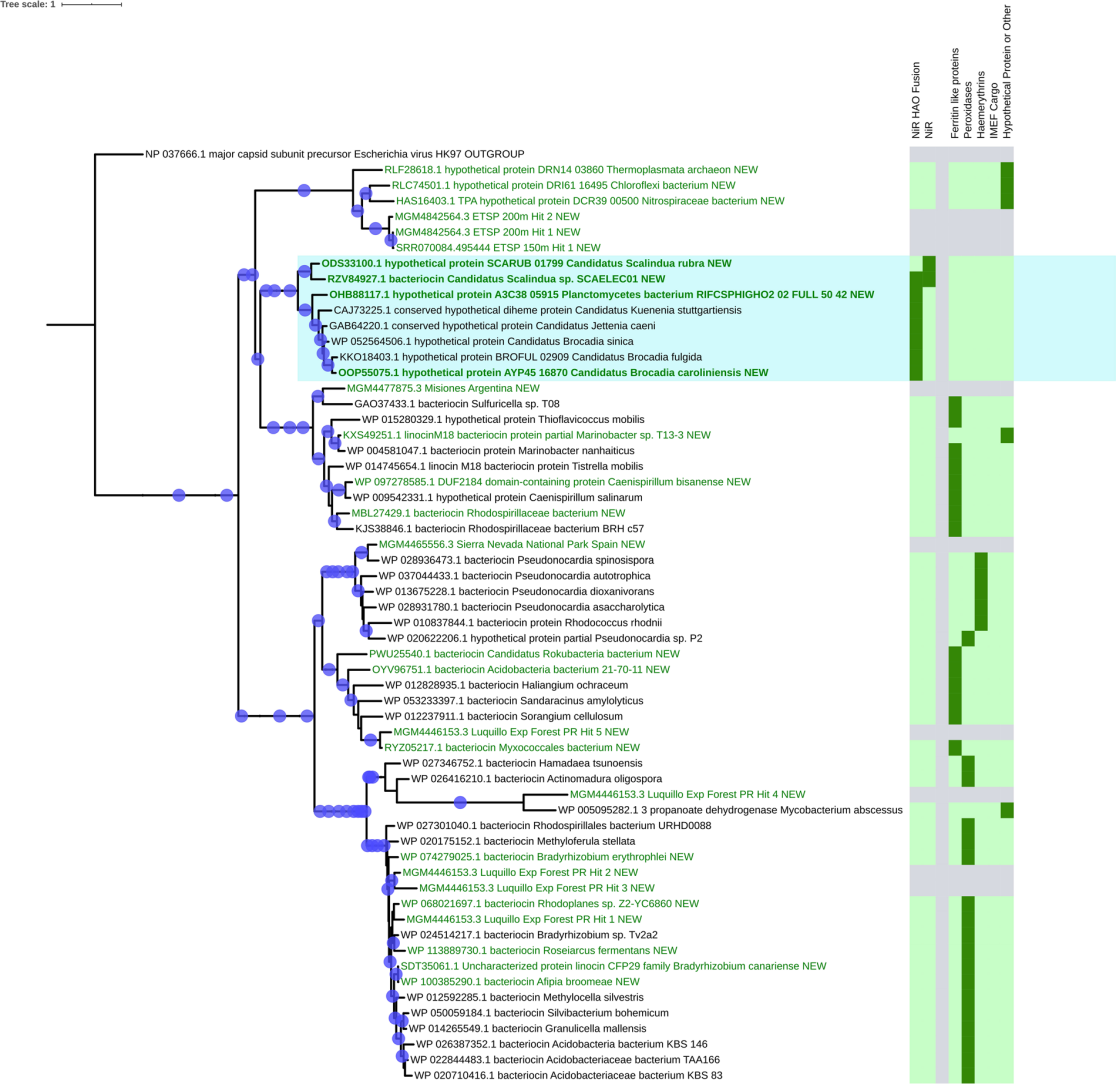
Newly discovered full-length encapsulin sequences cluster into clades with identical cargo protein types. Dark green rectangles indicate that a cargo protein of that type is encoded immediately next to the encapsulin in the tree, while light green rectangles show that the indicated cargo type is not present. Grey rectangles indicate encapsulins that are present on contigs that are too short to reveal other genes. All bootstrap values above 0.75 are displayed on the tree as blue circles. Newly discovered encapsulins are shown in green, while their known closest relatives are shown in black. Newly discovered anammox encapsulins are displayed in bold green text while the entire anammox clade is outlined in blue.

Additional *Scalindua* encapsulins were sought by searching environmental metagenomes with HMMER^24^. HMMER searches of five OMZ or anammox enrichment metagenomes, Stewart *et al*.^47^ (ETSP), van de Vossenberg *et al*.^29^ (anammox enrichment), Glass *et al*.^48^ (ETNP), Tsementzi *et al*.^49^ (ETNP), and Sun *et al*.^28^ (ETSP) with a HMM profile of the previously discovered 913 putative encapsulins^1^ (see Fig. 3 for metagenome locations and Table S1 for depths) and the above *Scalindua rubra* system as query, returned no additional *Scalindua* encapsulins. However, HMMER searches of Sun *et al*.^28^ (200 m depth) and Stewart *et al*.^47^ (150 m depth) returned three significant non-viral HMMER hits. These HMMER hits and the full-length sequences that contain them were confirmed to have homology to encapsulins through CD-searches (Tables S3 and S4). BLASTp searches with these HMMER sequences as queries returned that the best non-viral matches included proteins from a *Chloroflexi* bacterium, a Nitrospiraceae bacterium, and a *Thermoplasmata* archaeon (Table S5). None of these proteins had been previously reported as an encapsulin nanocompartment (Table S5). A close evolutionary relationship between these proteins and the three ETSP hits was also confirmed by phylogeny (Fig. 4).

To follow up on the potential of HMMER searches to discover novel encapsulins, HMMER searches were also conducted on additional metagenomes from environments other than OMZs including coastal waters and estuaries, tropical and temperate soils, the open ocean, and the human microbiome. These searches returned seven significant non-viral hits from three of the 23 surveyed metagenomes (Table S1). The greatest density of hits per sample site was found in tropical and temperate soils, as seen by the five hits found at the Luquillo Experimental Forest in Puerto Rico (Table S1). Each of these HMMER hits and the full-length sequences that contain them were confirmed to have homology to encapsulins by CD-search (Tables S3 and S4). BLASTp searches with these seven HMMER hits and their corresponding full-length sequences as queries showed that the best non-viral matches include 11 sequences not previously reported as encapsulins (Table S5). These 11 sequences include a DyP type peroxidase from *Rhodoplanes* sp. Z2-YC6860 as well as proteins from *Bradyrhizobium* (*B.*) *canariense*, *B*. *erythrophlei*, *Roseiarcus fermentans*, *Afipia broomae*, a “*Candidatus Rokubacteria*” bacterium, a Myxococcales bacterium, a Rhodospirillaceae bacterium, *Acidobacteria* bacterium 21-70-11, *Marinobacter* sp. T13-3, and *Caenispirillum bisanense*.

A HMMER search using encapsulin sequences as query could pick up a DyP type peroxidase cargo protein because some DyP type peroxidase encapsulins contain a unique fusion architecture where the encapsulin and cargo are fused together^5^. Examination of upstream and downstream genes from the *Rhodoplanes* sp. Z2-YC6860 DyP type peroxidase showed that the adjacent gene is a member of the Linocin M18 superfamily and thus an encapsulin. The identity of this system was further validated by the presence of the core cargo sequence in the cargo protein.

An important question not yet considered is if the full-length sequences that enclose the HMMER hits are themselves new encapsulins. The two pieces of information that are necessary to determine this are (1) how similar the full-length sequences are to each other and (2) how similar the full-length sequences are to the best matched BLASTp sequence. Pairwise sequence alignments performed using Jalview between full-length sequences revealed that all full-length sequences did not have percent identities above the 89% threshold (See Section S2 for a link to alignments).

Alignments between the full-length sequences and their best BLASTp matches showed that only the ETNP metagenome of Tsementzi *et al*.^49^ has a percent identity to its best BLASTp match that is above the 89% threshold (Table S5). As a result, ten of the 11 full-length sequences that contain significant non-viral HMMER hits are new encapsulins (only the Tsementzi *et al*.^49^ sequence is removed). In summary, four new encapsulins were discovered in Planctomycete bacterial genomes (Fig. 1a), ten in HMMER searches of environmental metagenomes, and 14 through BLASTp searches that sought to identify the above HMMER hits and the full-length sequences that contain them (Figs. 3 and 4).

A phylogenetic tree (Fig. 4) of the closest relatives of all the newly discovered full-length encapsulin sequences was constructed to further confirm the above BLASTp results and to try to identify the cargo protein type associated with each new encapsulin. This analysis showed that, for the most part, encapsulin sequences cluster by cargo protein type implying that evolution acts on the encapsulin and its cargo system as a unit. The only exceptions to this clustering are (1) in the topmost cluster of ferritin-like proteins, the cargo protein of *Marinobacter* sp. T13-3 was classified as a hypothetical protein/other, (2) in the haemerythrin cluster, *Pseudonocardia* sp. P2 possesses a peroxidase instead of a haemerythrin cargo, and (3) the cluster including hit 4 from the Luquillo Experimental Forest. This last cluster includes encapsulin sequences that are separated by large distances and a mixture of two peroxidase and one hypothetical protein/other cargo classifications. Due to the rarity of these exceptions, reasonable hypotheses can be proposed for the identity of the cargo proteins associated with each of the new encapsulins, except hit 4 from the Luquillo Experimental Forest, discovered in environmental metagenomes. The new encapsulins are predicted to include cargo proteins of four types: ferritin-like proteins, peroxidases, Haemerythrins (di-iron proteins that coordinate small molecules like O_2_), and "other" – proteins with different functions than those described above or hypothetical proteins with no predicted function (Table S6). Iron mineralizing encapsulins in *Firmicutes* (IMEF) type cargo proteins are only found in the *Firmicutes* lineage. As a result, no IMEF type cargo proteins were observed due to the lack of known *Firmicutes* sequences in the pruned tree.

## Discussion

Encapsulin bearing organisms often inhabit extreme environments^1^. The putative encapsulins found in *Scalindua rubra* (identified from a hypersaline brine interface), *Acidobacteria* bacterium 21-70-11 (isolated from mine wastewater), and *Thermoplasmata* archaeon (isolated from a marine hydrothermal sediment) all further support the idea that encapsulation could be particularly advantageous in extreme environments. In an extreme environment, organisms must adapt to harsh conditions such as extremes in temperatures, pressures, pH, and salinities. While the correlation of encapsulins to extreme environments does not prove that encapsulins are an adaptation to these environments, this hypothesis merits additional testing.

In addition to connections between environmental conditions and anammox encapsulins, there is some evidence of a connection between anammox encapsulins and the central anammox reaction elucidated by Kartal *et al*.^7^. This evidence rests on a proteomics study^11^ that showed encapsulin proteins are present within the anammoxosome as well as separate observations of small ~25 nm diameter electron and iron rich regions within the anammoxosome^50^. It has been previously hypothesized that these regions could be encapsulins based on the approximate size agreement between the electron rich regions and heterologously expressed heme containing *Kuenenia* encapsulins^1,51^. One proposed function of the anammoxosome is to create a proton gradient for ATP synthesis, analogous to a single eukaryotic mitochondrion^8^. This analogy is supported by an immunogold labelling study that localized ATP synthases to the anammoxosome membrane^52^. Since the only known function of the anammoxosome is energy generation, and encapsulins have been found within the anammoxosome, together these results give some credence to hypothesizing that encapsulins might play a role in the anammox process through the nitrite reductase piece of the cargo protein. However, direct proof of the role of encapsulins and their cargo proteins in the anammox reaction is still lacking.

Additional hypotheses for the function of anammox encapsulins have been formulated. The NiR-like section of the *K*. *stuttgartiensis* cargo protein is classified by CD-search as belonging to a family of NiR-like two domain laccases^22^. Laccases are enzymes that oxidize aromatic, particularly phenolic, compounds, while simultaneously reducing oxygen gas^53^. It is reasonable to hypothesize that aromatic oxidation instead of nitrite reduction could be the cargo protein’s function. In addition, many encapsulins are thought to be exported to the cell exterior^22^. If anammox encapsulins are exported and contain a functional laccase, then the function of anammox encapsulin cargo proteins may be to maintain an anaerobic environment around the outer cell membrane.

Although much progress has been made in the field of anammox biochemistry, many questions remain. These include explaining why anammox bacteria possess multiple copies of HAO*-*like proteins^15^ and tying the core anammox reaction elucidated by Kartal *et al*.^7^ to the metabolic flexibility^15,54–56^ that anammox organisms exhibit. Due to the HAO-like cargo proteins and cytochromes found in many encapsulins, a greater understanding of encapsulins may provide some clues to these outstanding questions.

It has become clear that anammox is a large contributor to marine fixed nitrogen loss. Anammox occurring in oxygen minimum zones and marine sediments is estimated to remove 70 Tg fixed N yr^−1^ ^57,58^. This large flux is performed almost entirely by members of the *Scalindua* genus. As a result, it is crucial to understand the fundamental biochemistry underlying this sink of fixed nitrogen. Research into the role of encapsulins in anammox biochemistry is a promising avenue towards addressing this need.

## Conclusion and Future Directions

While there are still relatively few experimentally characterized encapsulin systems, recent studies^1^ as well as this work provide additional evidence that encapsulin systems may be a widespread prokaryotic compartmentalization strategy with unappreciated diversity. This study discovered new anammox encapsulin systems in *Scalindua rubra*, *Scalindua* sp. SCAELEC01 167, *Brocadia caroliniensis*, as well as a new Planctomycete encapsulin from an aquifer metagenome. The two *Scalindua* species contain a different cargo protein architecture than previously known freshwater encapsulins, illustrating that even within an ecological guild encapsulins have enzymatic diversity. In addition to discovering new anammox encapsulins, metagenomic prospecting discovered twenty-four new encapsulins from a wide variety of environments. Phylogenetic analysis of these sequences as well as the new anammox sequences illustrate that, for the most part, encapsulins cluster by cargo protein type. This relationship provides evidence that encapsulins and their cargo evolve together and also allows the prediction of cargo proteins for encapsulins found in metagenomic samples. Lastly, as only a minute fraction of the MG-RAST database was surveyed, many additional encapsulins likely await discovery through future searches.

## Supplementary information

Supplementary Materials

Sequencing Files related to Scalindua rubra encapsulin expression

## Data Availability

A link to example code for all bioinformatics programs is included in the Supplemental Materials. All sequences discussed in this paper are available in public databases or are contained in the Supplemental Materials.
