# Supplementary material for: The Discovery of Twenty-Eight New Encapsulin Sequences, Including Three in Anammox Bacteria
Source: Sci Rep. 2019 Dec 27;9:20122. doi: 10.1038/s41598-019-56533-5 (PMC6934571; doi:10.1038/s41598-019-56533-5)
Supplement: Supplementary file 1 — Supplementary Materials [file 41598_2019_56533_MOESM1_ESM.docx]

**The Discovery of Twenty-Eight New Encapsulin Sequences,**

**Including Three in Anammox Bacteria**

John C. Tracey^1*^, Maricela Coronado^1^, Tobias W. Giessen^2, 3, 4^, Maggie C.Y. Lau^1, 5^,

Pamela A. Silver^3, 4^, and Bess B. Ward^1^

^1^ Princeton University, Department of Geosciences, Guyot Hall, Princeton, NJ 08544

^2^ Department of Biomedical Engineering, University of Michigan, Ann Arbor, MI 48109

^3^ Harvard Medical School, Department of Systems Biology, Boston, MA 02115

^4^ Wyss Institute for Biologically Inspired Engineering, 3 Blackfan Circle, Boston, MA 02115

^5^ Institute of Deep-Sea Science and Engineering, Chinese Academy of Sciences, Sanya, Hainan, China

**Supplemental Information**

**S1: Sequence of *S. rubra* Encapsulin and Multi-Copper Oxidase**

*Scalindua rubra* encapsulin with different start codons in bold:

>ODS33100.1 hypothetical protein SCARUB_01799 [“*Candidatus Scalindua rubra”*]

**M**GSNDASSFFLEVPLTHEQLEL**M**KRTVSRSARGHLVGRKFIKVYGPIGAGAQSVTWDTFLPPVMANIDLLGEAEQQPVHTQRRAILNIPQIYKDFILYGRDIAASHDHDSSENHNYPSHDRGHNHNHHGCICMPLDLSALVNAVVQCAKKEDDMIFSGLPEMGIPGLTNVEGRNIQPMKDWSILGNGFQDVVEAAQKLTDVGFYGPYAMVMSPHLYALLHRVYEKTGVLEIKSIKALIKGGVFQSSVLKSDVAIVVAMDQSNMDLVIGQDMRVSYWGPENLNHRFRVWESAVLRIKCPQAICTIEP

Enc 1:

**M**GSNDASSFFLEVPLTHEQLEL**M**KRTVSRSARGHLVGRKFIKVYGPIGAGAQSVTWDTFLPPVMANIDLLGEAEQQPVHTQRRAILNIPQIYKDFILYGRDIAASHDHDSSENHNYPSHDRGHNHNHHGCICMPLDLSALVNAVVQCAKKEDDMIFSGLPEMGIPGLTNVEGRNIQPMKDWSILGNGFQDVVEAAQKLTDVGFYGPYAMVMSPHLYALLHRVYEKTGVLEIKSIKALIKGGVFQSSVLKSDVAIVVAMDQSNMDLVIGQDMRVSYWGPENLNHRFRVWESAVLRIKCPQAICTIEP

Enc 2:

**M**KRTVSRSARGHLVGRKFIKVYGPIGAGAQSVTWDTFLPPVMANIDLLGEAEQQPVHTQRRAILNIPQIYKDFILYGRDIAASHDHDSSENHNYPSHDRGHNHNHHGCICMPLDLSALVNAVVQCAKKEDDMIFSGLPEMGIPGLTNVEGRNIQPMKDWSILGNGFQDVVEAAQKLTDVGFYGPYAMVMSPHLYALLHRVYEKTGVLEIKSIKALIKGGVFQSSVLKSDVAIVVAMDQSNMDLVIGQDMRVSYWGPENLNHRFRVWESAVLRIKCPQAICTIEP

Multi-Copper Oxidase with His-Tag:

MKMTISIFKWHTKLKHIALPTVLVVLTLLLLRNIAKSNEEWELGLQVKTPEIESKEEVKFEIEAKQIPLEIRPGIFFDAWAYGLKDQPPTVPGPTIHVKEGTKVKIHFTNKLSVPASIHPHGVKYTIKHDGAHLAGNPNTIVLPGESRTYEWDTDGTPGTWVYHTHAFEFGGEKGLEKGLFGPIIVDPKGTQSPPDKEFIVFLTTYKIGDKTFEAFNDKSGEAAFFQGDTTAFPGEVFHAKVGEKVRFHLVNADSEEMHTFHIHGHRWKNQRGGELIDNVSLGPFTMYTLDIIAGEDVGQGNWMVHCHFGSHMMMGMFGILAVRGSGSGHHHHHH

**S2: Example Bioinformatics Code:**

For all bioinformatics code, sequences of significant HMMER results, sequences of all new encapsulins described by this paper, and alignments to determine the similarity of the HMMER hits please see:

<https://github.com/jtracey1227/Encapsulins_metagenomes>

**Table S1: Description of Metagenomes used for HMMER Searches**

| **MG RAST**  **or SRA ID** | **Biome** | **Location** | **Study / Principle Investigator** | **Non-viral HMMER Hits** | **New Encapsulins** |
| --- | --- | --- | --- | --- | --- |
|  |  |  |  |  |  |
| mgm4842562.3 | OMZ | ETSP – 40m | Xin Sun | 0 | 0 |
| mgm4842563.3 | OMZ | ETSP - 80m | Xin Sun | 0 | 0 |
| mgm4842564.3 | OMZ | ETSP - 200m | Xin Sun | 2 | **5** |
| mgm4842565.3 | OMZ | ETSP - 300m | Xin Sun | 0 | 0 |
| N/A – obtained from author. | Anammox enrichment | Swedish fjord | van de Vossenberg *et al.,* 2013 | 0 | 0 |
| SRR064446 | OMZ | ETSP – 85m DNA | Stewart *et al.,* 2012 | 0 | 0 |
| SRR064447 | OMZ | ETSP – 85m cDNA | Stewart *et al.,* 2012 | 0 | 0 |
| SRR064448 | OMZ | ETSP – 110m - DNA | Stewart *et al.,* 2012 | 0 | 0 |
| SRR064449 | OMZ | ETSP – 110m - cDNA | Stewart *et al.,* 2012 | 0 | 0 |
| SRR064450 | OMZ | ETSP – 200m - DNA | Stewart *et al.,* 2012 | 0 | 0 |
| SRR064451 | OMZ | ETSP – 200m - cDNA | Stewart *et al.,* 2012 | 0 | 0 |
| SRR070082 | OMZ | ETSP – 200m - DNA | Stewart *et al.,* 2012 | 0 | 0 |
| SRR070083 | OMZ | ETSP – 80m - DNA | Stewart *et al.,* 2012 | 0 | 0 |
| SRR070084 | OMZ | ETSP – 150m - DNA | Stewart *et al.,* 2012 | 1 | **1** |
| SRR304668 | OMZ | ETSP – 500m - DNA | Stewart *et al.,* 2012 | 0 | 0 |
| SRR304683 | OMZ | ETSP – 800m - DNA | Stewart *et al.,* 2012 | 0 | 0 |
| SRR304673 | OMZ | ETSP – 110m - DNA | Stewart *et al.,* 2012 | 0 | 0 |
| SRR304680 | OMZ | ETSP – 110m - DNA | Stewart *et al.,* 2012 | 0 | 0 |
| Obtained assemblies from Xin Sun | ETNP – 68m | ETNP – 68m | Tsementzi *et al.,* 2016 | 1 | 0 |
| Obtained assemblies from Xin Sun | OMZ | ETNP – 120m | Tsementzi *et al.,* 2016 | 0 | 0 |
| Obtained assemblies from Xin Sun | OMZ | ETNP | Glass *et al.,* 2015 | 0 | 0 |
|  |  |  |  |  |  |
|  |  |  |  |  |  |
| mgm4441593.3 | ETNP- coastal surface | Dirty Rock, Cocos Island, Costa Rica | Global Ocean Sampling Expedition | 0 | 0 |
| mgm4441661.3 | ETNP - open ocean surface | 30 miles off Cocos Island, Costa Rica | Global Ocean Sampling Expedition | 0 | 0 |
| mgm4441591.3 | ETNP - coastal surface | Gulf of Panama | Global Ocean Sampling Expedition | 0 | 0 |
| mgm4441658.3 | Estuary | Delaware Bay | Global Ocean Sampling Expedition | 0 | 0 |
| mgm4441582.3 | Coastal Bay | Bay of Fundy, Nova Scotia | Global Ocean Sampling Expedition | 0 | 0 |
| mgm4441576.3 | Open ocean | Sargasso Sea, Bermuda | Global Ocean Sampling Expedition | 0 | 0 |
| mgm4720058.3 | Coastal surface waters | Bay of Bengal off Sri Lanka | Ocean Sampling Day | 0 | 0 |
| mgm4719986.3 | Sulfidic, low O_2_ | Black Sea | Ocean Sampling Day | 0 | 0 |
| mgm4719978.3 | Coastal Sea | Gulf of Aqaba, Red Sea | Ocean Sampling Day | 0 | 0 |
| mgm4719975.3 | Coastal Sea | Skaggerak, North Sea | Ocean Sampling Day | 0 | 0 |
| mgm4719974.3 | Coastal | Coastal off  Savannah, GA | Ocean Sampling Day | 0 | 0 |
| mgm4719971.3 | Coastal | 10 miles off Molokai, HI | Ocean Sampling Day | 0 | 0 |
| mgm4719951.3 | Arctic | Sea of Greenland | Ocean Sampling Day | 0 | 0 |
| mgm4722290.3 | Open ocean | 100 miles North of Antarctic Peninsula | Tara Oceans Southern Ocean | Could not assemble | 0 |
| mgm4461588.3 | Coastal OMZ surface | Sulfidic event in Peruvian OMZ | Schunck *et al*., 2013 | 0 | 0 |
| mgm4446153.3 | Tropical rain forest soil | Luquillo Exp. Forest, Puerto Rico | T. Hazen, Lawrence Berkeley Natl. Lab | 5 | **13** |
| mgm4478222.3 | Soybean rhizosphere | 50 miles W of Sinop, Brazil | L. W. Mendes, Center for Nuclear Energy in Ag. CENA-USP | 0 | 0 |
| mgm4465556.3 | Oak rhizosphere | Chaparral and Temperate Forest, Spain | Manuel Fernandez-Lopez | 1 | **1** |
| mgm4575383.3 | Coniferous forest | Fairbanks, Alaska | Noah Fierer, University of Colorado | 0 | 0 |
| mgm4477875.3 | Subtropical forest | Misiones, Argentina | Noah Fierer, University of Colorado | 1 | **4** |
| mgm4472711.3 | Human microbiome | Human nostrils | Human microbiome project, NIH | 0 | 0 |
| mgm4472547.3 | Human microbiome | Human nostrils | Human microbiome project, NIH | 0 | 0 |
| N/A | Saltwater cave aquifer | Dominican Republic | Zena Cardman and Jennifer Macalady | 0 | 0 |

**Table S1:** Description of metagenomes used for HMMER searches. Listed from left to right are the MG – RAST or NCBI accession numbers for each metagenome, the metagenome’s biome and location, the investigators who created the metagenome, the number of significant non-viral hits found in HMMER searches of each metagenome, and the number of new encapsulins. The number of new encapsulins discovered at each site includes both (1) HMMER sequences deemed to be novel, non-viral encapsulins and (2) new, non-viral encapsulins found among the top three BLASTp matches to the HMMER sequences and the full-length encapsulin. Rows shadowed dark blue indicate locations where new encapsulins were discovered. Rows shadowed light blue indicate locations where significant non-viral HMMER hits were observed, but no new encapsulins.

**Table S2: Summary of Anammox Encapsulin BLASTp Search Results: *Scalindua* only**

| **Query** | **Name of *Scalindua* hit** | **Bit Score** | **Query Coverage** | **E-value** | **Percent Identity** | **Accession # of *Scalindua* hit** |
| --- | --- | --- | --- | --- | --- | --- |
| *Kuenenia* encapsulin | Hypothetical Protein SCARUB 01799 | 294 | 74% | 6*10^-95^ | 51% | ODS33100.1 |
| *B. sinica* encapsulin | SCARUB 01799 | 296 | 71% | 9*10^-96^ | 52% | ODS33100.1 |
| *B. fulgida* encapsulin | SCARUB 01799 | 288 | 71% | 1*10^-92^ | 50% | ODS33100.1 |
| *J. caeni.* encapsulin | SCARUB 01799 | 307 | 71% | 7*10^-100^ | 53% | ODS33100.1 |
| ***Kuenenia* NiR/HAO** |  |  |  |  |  |  |
| 1 | HAO “*Candidatus Scalindua brodae”* | 523 | 58% | 2*10^-174^ | 50% | KHE92011.1 |
| 2 | HAO “*Candidatus Scalindua rubra”* | 517 | 57% | 3*10^-172^ | 50% | ODS34455.1 |
| 3 | HAO “*Candidatus Scalindua rubra”* | 517 | 57% | 6*10^-172^ | 50% | ODS30984.1 |
| 4 | HAO “*Candidatus Scalindua rubra”* | 515 | 58% | 1*10^-170^ | 50% | ODS34784.1 |
| 5 | HAO “*Candidatus Scalindua brodae”* | 466 | 57% | 2*10^-151^ | 46% | KHE90580.1 |
| 6 | HAO “*Candidatus Scalindua brodae”* | 330 | 37% | 2*10^-102^ | 48% | KHE90393.1 |
| 7 | MCO “*Candidatus Scalindua rubra*” | 308 | 31% | 2*10^-94^ | 58% | ODS33099.1 |
| 8 | HAO “*Candidatus Scalindua brodae”* | 218 | 57% | 6*10^-59^ | 32% | KHE93468.1 |
| ***B. sinica* NiR/HAO** |  |  |  |  |  |  |
| 1 | HAO “*Candidatus Scalindua brodae”* | 568 | 58% | 0 | 52% | KHE92011.1 |
| 2 | HAO “*Candidatus Scalindua rubra”* | 547 | 58% | 0 | 51% | ODS34784.1 |
| 3 | HAO “*Candidatus Scalindua rubra”* | 545 | 56% | 0 | 50% | ODS30984.1 |
| 4 | HAO “*Candidatus Scalindua rubra”* | 544 | 56% | 0 | 50% | ODS34455.1 |
| 5 | HAO “*Candidatus Scalindua brodae”* | 479 | 56% | 2*10^-156^ | 45% | KHE90580.1 |
| 6 | MCO “*Candidatus Scalindua rubra”* | 372 | 30% | 2*10^-118^ | 64% | ODS33099.1 |
| 7 | HAO “*Candidatus Scalindua brodae”* | 345 | 34% | 7*10^-108^ | 51% | KHE90393.1 |
| 8 | HAO “*Candidatus Scalindua brodae”* | 222 | 55% | 1*10^-60^ | 31% | KHE93468.1 |
| ***J. caeni* NiR/HAO** |  |  |  |  |  |  |
| 1 | HAO “*Candidatus Scalindua rubra”* | 536 | 61% | 9*10^-179^ | 50% | ODS34784.1 |
| 2 | HAO “*Candidatus Scalindua brodae”* | 530 | 60% | 5*10^-177^ | 49% | KHE92011.1 |
| 3 | HAO “*Candidatus Scalindua rubra”* | 510 | 61% | 9*10^-170^ | 46% | ODS34455.1 |
| 4 | HAO “*Candidatus Scalindua rubra”* | 510 | 61% | 2*10^-169^ | 46% | ODS30984.1 |
| 5 | HAO “*Candidatus Scalindua brodae”* | 473 | 59% | 3*10^-154^ | 46% | KHE90580.1 |
| 6 | MCO “*Candidatus Scalindua rubra”* | 354 | 31% | 5*10^-112^ | 62% | ODS33099.1 |
| 7 | HAO “*Candidatus Scalindua brodae”* | 325 | 37% | 1*10^-100^ | 47% | KHE90393.1 |
| 8 | HAO “*Candidatus Scalindua brodae”* | 219 | 58% | 1*10^-59^ | 30% | KHE93468.1 |
| ***B. fulgida* NiR/HAO** |  |  |  |  |  |  |
| 1 | HAO “*Candidatus Scalindua rubra”* | 540 | 60% | 0 | 50% | ODS34455.1 |
| 2 | HAO “*Candidatus Scalindua rubra”* | 540 | 61% | 0 | 49% | ODS30984.1 |
| 3 | HAO “*Candidatus Scalindua brodae”* | 537 | 60% | 6*10^-180^ | 48% | KHE92011.1 |
| 4 | HAO “*Candidatus Scalindua rubra”* | 531 | 60% | 5*10^-177^ | 49% | ODS34784.1 |
| 5 | HAO “*Candidatus Scalindua brodae”* | 488 | 59% | 3*10^-160^ | 47% | KHE90580.1 |
| 6 | HAO “*Candidatus Scalindua brodae”* | 343 | 38% | 1*10^-107^ | 49% | KHE90393.1 |
| 7 | MCO “*Candidatus Scalindua rubra”* | 362 | 31% | 6*10^-90^ | 62% | ODS33099.1 |
| 8 | HAO “*Candidatus Scalindua brodae”* | 222 | 58% | 1*10^-60^ | 31% | KHE93468.1 |

**Table S2:** Results from BLASTp searches that used the freshwater anammox encapsulin and cargo proteins as queries of the nr database. Results for each search using the freshwater anammox encapsulin as the query are highlighted in blue. Hypothetical protein SCARUB 01799 is the newly discovered *Scalindua rubra* encapsulin. Results for searches using the freshwater anammox fusion cargo protein as query that returned the multi-copper oxidase as hits are shadowed in green. Searches using the freshwater cargo proteins as query returned many *Scalindua* HAO proteins; however, these HAO proteins are either from different species or not present on the same contig as the encapsulin.

**Table S3: Verification that Non-viral HMMER Hits are Encapsulins by CD – Search**

**HMMER Hits Alone as Query**

| **Metagenome**  **Searched** | **HMMER**  **Hit #** | **Linocin M18 Protein Superfamily Found?** | **Amino Acid Interval Over Which**  **Similarity Occurs** | **E - value** |
| --- | --- | --- | --- | --- |
| **Xin Sun 200m** | 1 | Yes | 1 - 141 out of 218 | 6.87*10^-9^ |
|  | 2 | Yes | 1 - 129 out of 130 | 2.02*10^-8^ |
| **Stewart 150m** | 1 | Yes | 1 - 51 out of 55 | 7.68*10^-9^ |
| **Tsementzi *et al.,* 2016** | 1 | Yes | 1 -56 out of 56 | 7.76*10^-25^ |
| **Luquillo Exp. Forest** | 1 | Yes | 3 - 132 out of 132 | 1.2*10^-60^ |
|  | 2 | Yes | 1 - 115 out of 115 | 4.68*10^-50^ |
|  | 3 | Yes | 1 -124 out of 124 | 1.2*10^-37^ |
|  | 4 | Yes | 1 -37 out of 53 | 4.36*10 ^-9^ |
|  | 5 | Yes | 1 - 57 out of 57 | 6.91*10^-18^ |
| **Oak rhizosphere, Spain** | 1 | Yes | 1 - 155 out of 155 | 1.02*10^-59^ |
| **Misiones, Argentina** | 1 | Yes | 1 - 94 out of 95 | 3.56*10^-24^ |

**Table S3:** All significant non-viral HMMER hits are identified by CD search as encapsulins. CD search of the CDD v3.16 database was carried out using the default parameters. The interval over which homology to the Linocin M18 protein superfamily occurs is reported out of the total length of the HMMER hit. The E-value refers to the probability that the homology detected by CD search is due to chance.

**Table S4: Verification that Non-viral HMMER Hits are Encapsulins by CD – Search**

**Full-length Encapsulin Recovered from Metagenome as Query**

| **Metagenome**  **Searched** | **HMMER**  **Hit #** | **Linocin M18 Protein Superfamily Found?** | **Amino Acid Interval Over Which**  **Similarity Occurs** | **E - value** |
| --- | --- | --- | --- | --- |
| **Xin Sun 200m** | 1 | Yes | 50 - 281 out of 285 | 3.18*10^-9^ |
|  | 2 | Yes | 43 - 171 out of 172 | 1.51*10^-8^ |
| **Stewart 150m** | 1 | Yes | 53 - 113 out of 126 | 3.70*10^-8^ |
| **Tsementzi *et al.,* 2016** | 1 | Yes | 1 -59 out of 59 | 5.35*10^-28^ |
| **Luquillo Exp. Forest** | 1 | Yes | 19 - 149 out of 149 | 5.99*10^-62^ |
|  | 2 | Yes | 1 - 117 out of 117 | 9.40*10^-53^ |
|  | 3 | Yes | 1 -126 out of 139 | 3.35*10^-38^ |
|  | 4 | Yes | 2 -39 out of 78 | 4.91*10 ^-10^ |
|  | 5 | Yes | 1 - 62 out of 67 | 5.21*10^-20^ |
| **Oak rhizosphere, Spain** | 1 | Yes | 15 - 161 out of 161 | 8.69*10^-56^ |
| **Misiones, Argentina** | 1 | Yes | 1 - 100 out of 104 | 4.80*10^-25^ |

**Table S4:** The genes containing each significant non-viral HMMER hit listed in Table S3 were identified by CD search as encapsulins. CD search of the CDD v3.16 database was carried out using the default parameters. The interval over which homology to the Linocin M18 protein superfamily occurs is reported out of the total length of the full length sequence from the metagenomes. The E-value refers to the probability that the homology detected by CD search is due to chance.

**Table S5: Identifying Encapsulins by BLASTp Search**

| **Metagenome**  **Searched** | **HMMER**  **Hit #** | **Top Three Matches** | **Species Name** | **Percent Identity** | **E - value** | **New Encapsulin?** |
| --- | --- | --- | --- | --- | --- | --- |
| **Xin Sun 200m** |  |  |  |  |  |  |
| HMMER Hit | 1 | RLC74501.1 | *Chloroflexi* bacterium | 45% | 1*10^-58^ | Yes |
| HMMER Hit | 1 | OQY11132.1 | *Desulfobacteraceae* bacterium 4572_19 | 46% | 1*10^-55^ | No (Viral) |
| HMMER Hit | 1 | RLF28618.1 | *Thermoplasmata archaeon* | 37% | 2*10^-40^ | Yes |
| Full Length | 1 | RLC74501.1 | *Chloroflexi* bacterium | 43% | 5*10^-70^ | Yes |
| Full Length | 1 | HAS16403.1 | Nitrospiraceae bacterium | 42% | 2*10^-64^ | Yes |
| Full Length | 1 | OQY11132.1 | *Desulfobacteraceae* bacterium 4572_19 | 43% | 1*10^-59^ | No |
|  |  |  |  |  |  |  |
| **Xin Sun 200m** |  |  |  |  |  |  |
| HMMER Hit | 2 | OQY11132.1 | *Desulfobacteraceae* bacterium 4572_19 | 52% | 3*10^-34^ | No |
| HMMER Hit | 2 | RLC74501.1 | *Chloroflexi* bacterium | 47% | 4*10^-32^ | Yes |
| HMMER Hit | 2 | RLF28618.1 | *Thermoplasmata archaeon* | 47% | 1*10^-29^ | Yes |
| Full Length | 2 | RLC74501.1 | *Chloroflexi* bacterium | 42% | 9*10^-37^ | Yes |
| Full Length | 2 | OQY11132.1 | *Desulfobacteraceae* bacterium 4572_19 | 49% | 6*10^-34^ | No |
| Full Length | 2 | HAS16403.1 | Nitrospiraceae bacterium | 39% | 7*10^-32^ | Yes |
|  |  |  |  |  |  |  |
| **Stewart 150m** |  |  |  |  |  |  |
| HMMER Hit | 1 | OQY11132.1 | Desulfobacteraceae bacterium 4572_19 | 53% | 3*10^-7^ | No |
| HMMER Hit | 1 | STY61410.1 | *Mannheimia haemolytica* | 40% | 2*10^-4^ | No (Viral) |
| HMMER Hit | 1 | WP_088197229.1 | *Sinorhizobium meliloti* | 47% | 2*10^-4^ | No (Viral) |
| Full Length | 1 | RLC74501.1 | *Chloroflexi* bacterium | 46% | 1*10^-29^ | Yes |
| Full Length | 1 | HAS16403.1 | Nitrospiraceae bacterium | 48% | 2*10^-29^ | Yes |
| Full Length | 1 | RLF28618.1 | *Thermoplasmata archaeon* | 45% | 1*10^-26^ | Yes |
|  |  |  |  |  |  |  |
| **Luquillo Exp. Forest** |  |  |  |  |  |  |
| HMMER Hit | 1 | WP_068021697.1 | *Rhodoplanes* sp. Z2-YC6860  Dyp type peroxidase family protein | 87% | 1*10^-77^ | Yes |
| HMMER Hit | 1 | WP_024514217.1 | *Bradyrhizobium* sp. Tv2a-2 | 85% | 2*10^-74^ | No (previously described) |
| HMMER Hit | 1 | SDT35061.1 | *Bradyrhizobium canariense* | 83% | 3*10^-73^ | Yes |
| Full Length | 1 | WP_068021697.1 | *Rhodoplanes* sp. Z2-YC6860  Dyp type peroxidase family protein | 87% | 3*10^-78^ | Yes |
| Full Length | 1 | WP_024514217.1 | *Bradyrhizobium* sp. Tv2a-2 | 86% | 8*10^-75^ | No (previously described) |
| Full Length | 1 | SDT35061.1 | *Bradyrhizobium canariense* | 82% | 3*10^-73^ | Yes |
|  |  |  |  |  |  |  |
|  |  |  |  |  |  |  |
|  |  |  |  |  |  |  |
| **Luquillo Exp. Forest** |  |  |  |  |  |  |
| HMMER Hit | 2 | WP_074279025.1 | *Bradyrhizobium erythrophlei* | 82% | 7*10 ^-61^ | Yes |
| HMMER Hit | 2 | WP_024514217.1 | *Bradyrhizobium* sp. Tv2a-2 | 81% | 1*10^-60^ | No |
| HMMER Hit | 2 | WP_113889730.1 | *Roseiarcus fermentans* | 82% | 4*10^-60^ | Yes |
| Full Length | 2 | WP_074279025.1 | *Bradyrhizobium erythrophlei* | 82% | 9*10 ^-63^ | Yes |
| Full Length | 2 | WP_024514217.1 | *Bradyrhizobium* sp. Tv2a-2 | 81% | 1*10^-62^ | No |
| Full Length | 2 | WP_113889730.1 | *Roseiarcus fermentans* | 82% | 5*10^-62^ | Yes |
|  |  |  |  |  |  |  |
| **Luquillo Exp Forest** |  |  |  |  |  |  |
| HMMER Hit | 3 | WP_074279025.1 | Bradyrhizobium erythrophei | 71% | 4*10 ^-51^ | Yes |
| HMMER Hit | 3 | WP_068021697.1 | *Rhodoplanes* sp. Z2-YC6860  Dyp type peroxidase family protein | 70% | 1*10 ^-50^ | Yes |
| HMMER Hit | 3 | WP_100385290.1 | *Afipia broomeae* | 69% | 1*10 ^-50^ | Yes |
| Full Length | 3 | WP_074279025.1 | Bradyrhizobium erythrophei | 71% | 5*10 ^-52^ | Yes |
| Full Length | 3 | WP_100385290.1 | *Afipia broomeae* | 70% | 1*10 ^-51^ | Yes |
| Full Length | 3 | WP_068021697.1 | *Rhodoplanes* sp. Z2-YC6860  Dyp type peroxidase family protein | 70% | 2*10 ^-51^ | Yes |
|  |  |  |  |  |  |  |
| **Luquillo Exp Forest** |  |  |  |  |  |  |
| HMMER Hit | 4 | WP_024514217.1 | *Bradyrhizobium* sp. Tv2a-2 | 81% | 3*10 ^-18^ | No |
| HMMER Hit | 4 | WP_027301040.1 | *Rhodospirillales* bacterium URHD0088 | 77% | 4*10 ^-18^ | No (previously described) |
| HMMER Hit | 4 | WP_113889730.1 | *Roseiarcus fermentans* | 79% | 3*10 ^-17^ | Yes |
| Full Length | 4 | WP_027301040.1 | *Rhodospirillales* bacterium URHD0088 | 76% | 1*10 ^-21^ | No |
| Full Length | 4 | WP_024514217.1 | *Bradyrhizobium* sp. Tv2a-2 | 79% | 5*10 ^-20^ | No |
| Full Length | 4 | WP_100385290.1 | *Afipia broomeae* | 78% | 1*10 ^-19^ | Yes |
|  |  |  |  |  |  |  |
| **Luquillo Exp Forest** |  |  |  |  |  |  |
| HMMER Hit | 5 | WP_053233397.1 | *Sandaracinus amylolyticus* | 62% | 5*10 ^-12^ | No (previously described) |
| HMMER Hit | 5 | PWU25540.1 | *“Candidatus Rokubacteria”* bacterium | 64% | 5*10 ^-11^ | Yes |
| HMMER Hit | 5 | OYV96751.1 | *Acidobacteria* bacterium 21-70-11 | 67% | 1*10 ^-09^ | Yes |
| Full Length | 5 | RYZ05217.1 | *Myxococcales* bacterium | 78% | 8*10 ^-20^ | Yes |
| Full Length | 5 | WP_053233397.1 | *Sandaracinus amylolyticus* | 63% | 2*10 ^-13^ | No |
| Full Length | 5 | WP_012237911.1 | *Sorangium cellulosum* | 60% | 3*10 ^-12^ | No (previously described) |
|  |  |  |  |  |  |  |
| **Tsementzi *et al.,* 2016** |  |  |  |  |  |  |
| HMMER Hit | 1 | WP_017980848.1 | *Alphaproteobacteria* multispecies protein | 100% | 3*10 ^-30^ | No (previously described) |
| HMMER Hit | 1 | WP_062127258.1 | *Sphingomonas melonis* | 96% | 5*10 ^-29^ | No (previously described) |
| HMMER Hit | 1 | PAV92542.1 | *Diploscapter pachys* | 100% | 1*10 ^-28^ | No  (likely bacterial contamination) |
| Full Length | 1 | WP_017980848.1 | *Alphaproteobacteria* multispecies protein | 100% | 5*10 ^-33^ | No (previously described) |
| Full Length | 1 | WP_062127258.1 | *Sphingomonas melonis* | 97% | 1*10 ^-31^ | No (previously described) |
| Full Length | 1 | PAV92542.1 | *Diploscapter pachys* | 100% | 4*10 ^-31^ | No  (likely bacterial contamination) |
|  |  |  |  |  |  |  |
| **Oak rhizosphere, Spain** |  |  |  |  |  |  |
| HMMER Hit | 1 | WP_028936473.1 | *Pseudonocardia spinosispora* | 79% | 3*10 ^-79^ | No (previously described) |
| HMMER Hit | 1 | WP_103381197.1 | *Pseudonocardia dioxanivorans* | 66% | 5*10 ^-65^ | No (previously described) |
| HMMER Hit | 1 | WP_013675228.1 | *Pseudonocardia dioxanivorans* | 66% | 2*10 ^-64^ | No (previously described) |
| Full Length | 1 | WP_028936473.1 | *Pseudonocardia spinosispora* | 79% | 6*10 ^-80^ | No (previously described) |
| Full Length | 1 | WP_103381197.1 | *Pseudonocardia dioxanivorans* | 67% | 2*10 ^-65^ | No (previously described) |
| Full Length | 1 | WP_028931780.1 | *Pseudonocardia asaccharolytica* | 64% | 7*10 ^-65^ | No (previously described) |
|  |  |  |  |  |  |  |
| **Misiones, Argentina** |  |  |  |  |  |  |
| HMMER Hit | 1 | WP_009542331.1 | *Caenispirillum salinarum* | 65% | 2*10 ^-34^ | No (previously described) |
| HMMER Hit | 1 | KXS49251.1 | *Marinobacter sp.* T13-3 | 59% | 7*10 ^-33^ | Yes |
| HMMER Hit | 1 | WP_097278585.1 | *Caenispirillum bisanense* | 61% | 2*10 ^-32^ | Yes |
| Full Length | 1 | WP_009542331.1 | *Caenispirillum salinarum* | 62% | 2*10 ^-35^ | No (previously described) |
| Full Length | 1 | MBL27429.1 | Rhodospirillaceae bacterium | 63% | 2*10 ^-34^ | Yes |
| Full Length | 1 | WP_097278585.1 | *Caenispirillum bisanense* | 59% | 2*10 ^-33^ | Yes |

**Table S5:**  BLASTp searches with each significant, non-viral HMMER sequence (rows marked HMMER Hit) and the full length encapsulin gene that contains the hit (Full Length) discovered fourteen new encapsulins. The nr database was searched with default parameters. The top three results of both the HMMER hit and full length searches are displayed with taxonomic information, percent identity to the query, and E-value. New encapsulins undetected by previous searches are colored in blue.

**Table S6: Proposed Cargo Protein Types for Newly Discovered Encapsulins in**

**Environmental Metagenomes**

| **Metagenome**  **Searched** | **HMMER**  **Hit #** | **Proposed Cargo Type** |
| --- | --- | --- |
| **Xin Sun 200m** | 1 | Other |
|  | 2 | Other |
| **Stewart 150m** | 1 | Other |
| **Luquillo Exp. Forest** | 1 | Peroxidase |
|  | 2 | Peroxidase |
|  | 3 | Peroxidase |
|  | 4 | Other |
|  | 5 | Ferritin like protein |
| **Oak rhizosphere, Spain** | 1 | Haemerythrin |
| **Misiones, Argentina** | 1 | Ferritin like protein |

**Table S6:** Proposed cargo protein types for newly discovered encapsulins in environmental metagenomes. Other indicates an encapsulin cargo protein that is a hypothetical protein without a currently known function.

**Figure S1: Novex Tris-Glycine Gel of *S. rubra* Enc and MCO Co-expression under One Promoter**

**
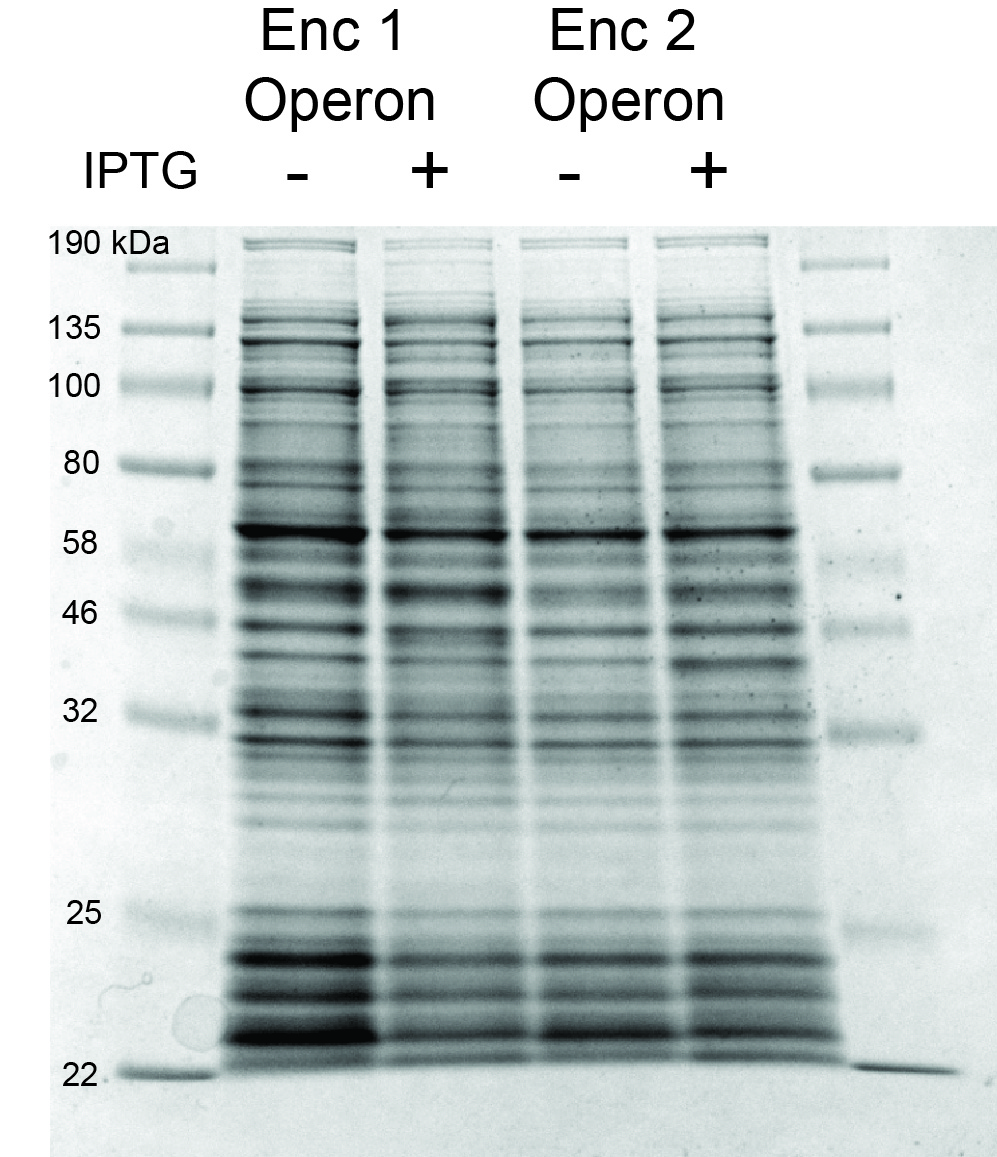
**

**Figure S1:** Co-expression of the *S. rubra* encapsulin and multi-copper oxidase under one promoter was unsuccessful. No response to IPTG induction at 34 kDa (encapsulin monomer MW) or 36 kDa (multi-copper oxidase MW) was observed. This result led us to place Enc 1, Enc 2, and the MCO under the control of separate promoters. This result is shown in Figure 2 and Figure S3 below.

**Figure S2: Uncropped Novex Tris-Glycine Gel of *S. rubra* Enc and MCO Co-expression under One Promoter**

**
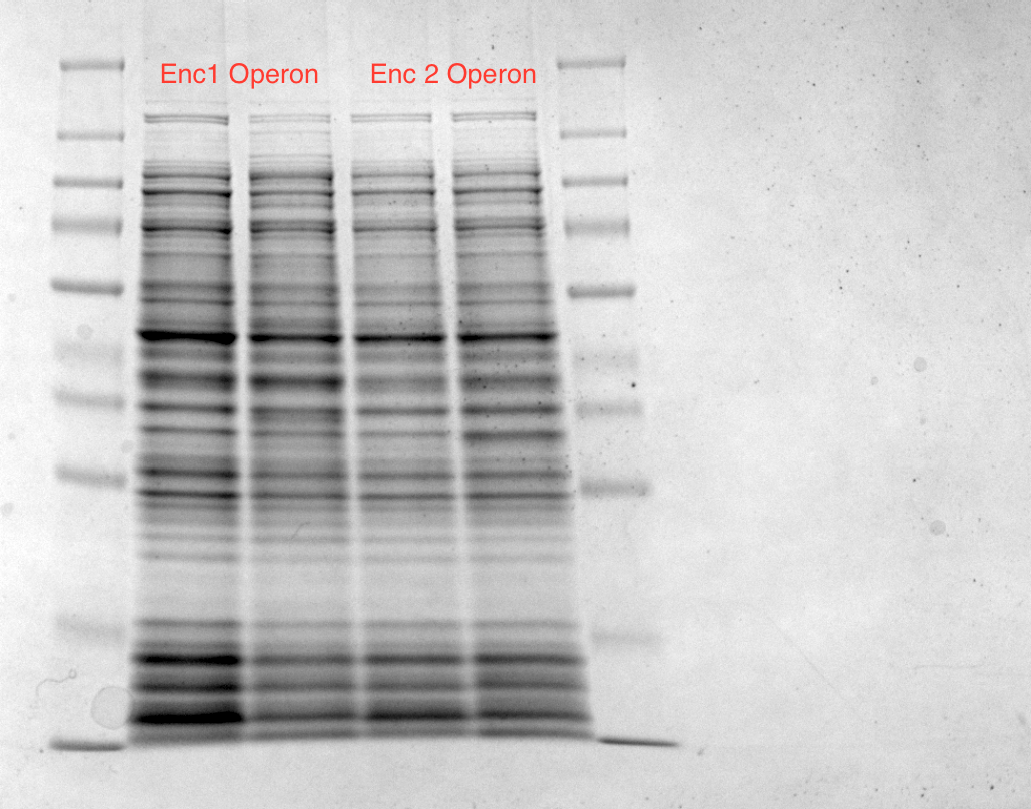
**

**Figure S2:** Uncropped image of the gel shown in Figure S1.

**Figure S3: Uncropped Novex Tris-Glycine gel of *E. coli* expression experiment seen in Figure 2**

**
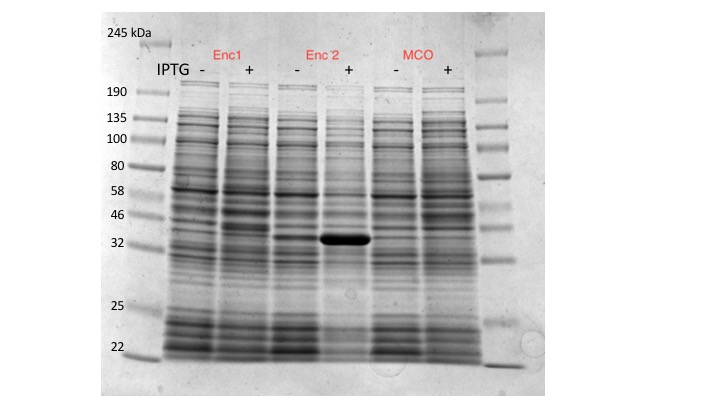
**

**Figure S3:** Only Enc 2 responds strongly to IPTG induction. Enc 1 and MCO are not expressed above background. Enc 1, Enc 2, and MCO were under the control of separate promoters.

**Figure S4: SDS -PAGE gel of purified Enc 2 protein**

**
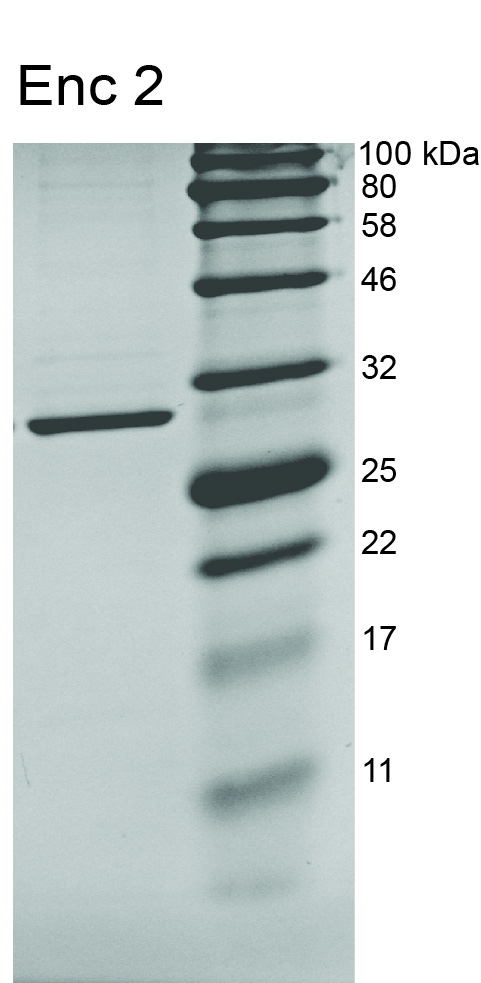
**

**Figure S4:** SDS PAGE gel electrophoresis showing the purified Enc 2 monomer protein. Although the molecular weight inferred from the gel is lower than the predicted weight of 34 kDa, this is not surprising because capsid proteins are known to run lower on SDS PAGE gels. In addition, molecular weights are heavily dependent on running conditions and are only approximated by SDS PAGE.

**Figure S5: Uncropped SDS -PAGE gel of purified Enc 2 protein**


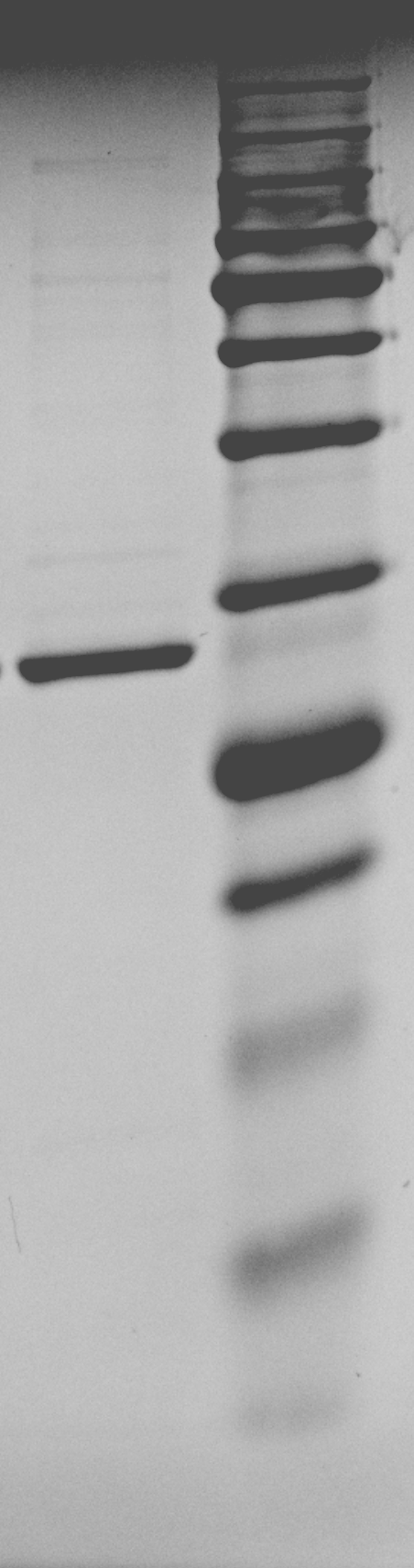


**Figure S5:** Uncropped image of the gel shown above in Figure S2.

**Figure S6: Uncropped TEM image of the *Scalindua rubra* encapsulin (Figure 2)**

**
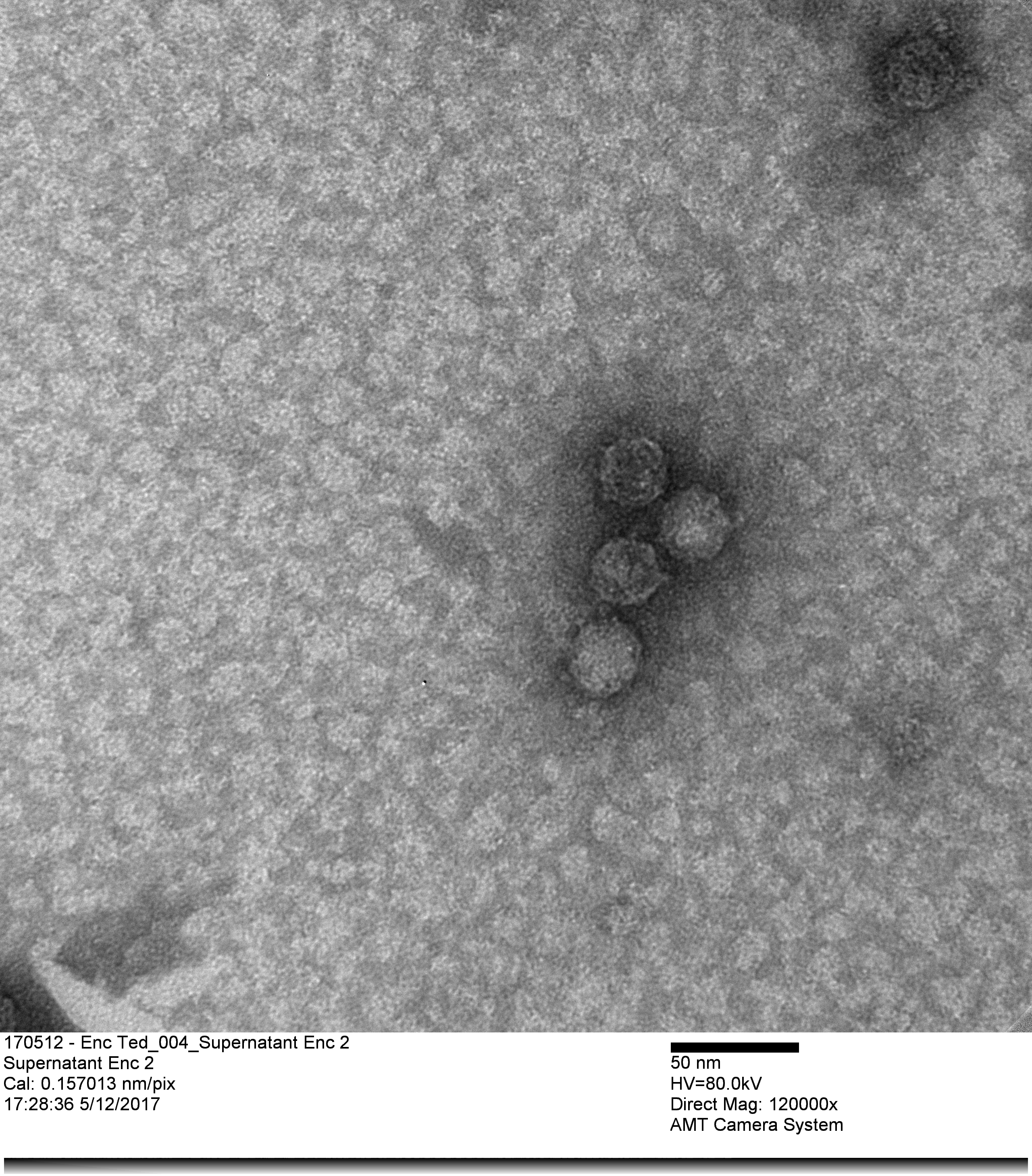
**

**Figure S6:** Uncropped TEM image of the purified, assembled *Scalindua rubra* encapsulin.

**Figure S7: Percent Identities of all Anammox and Planctomycete Encapsulin Systems**

**
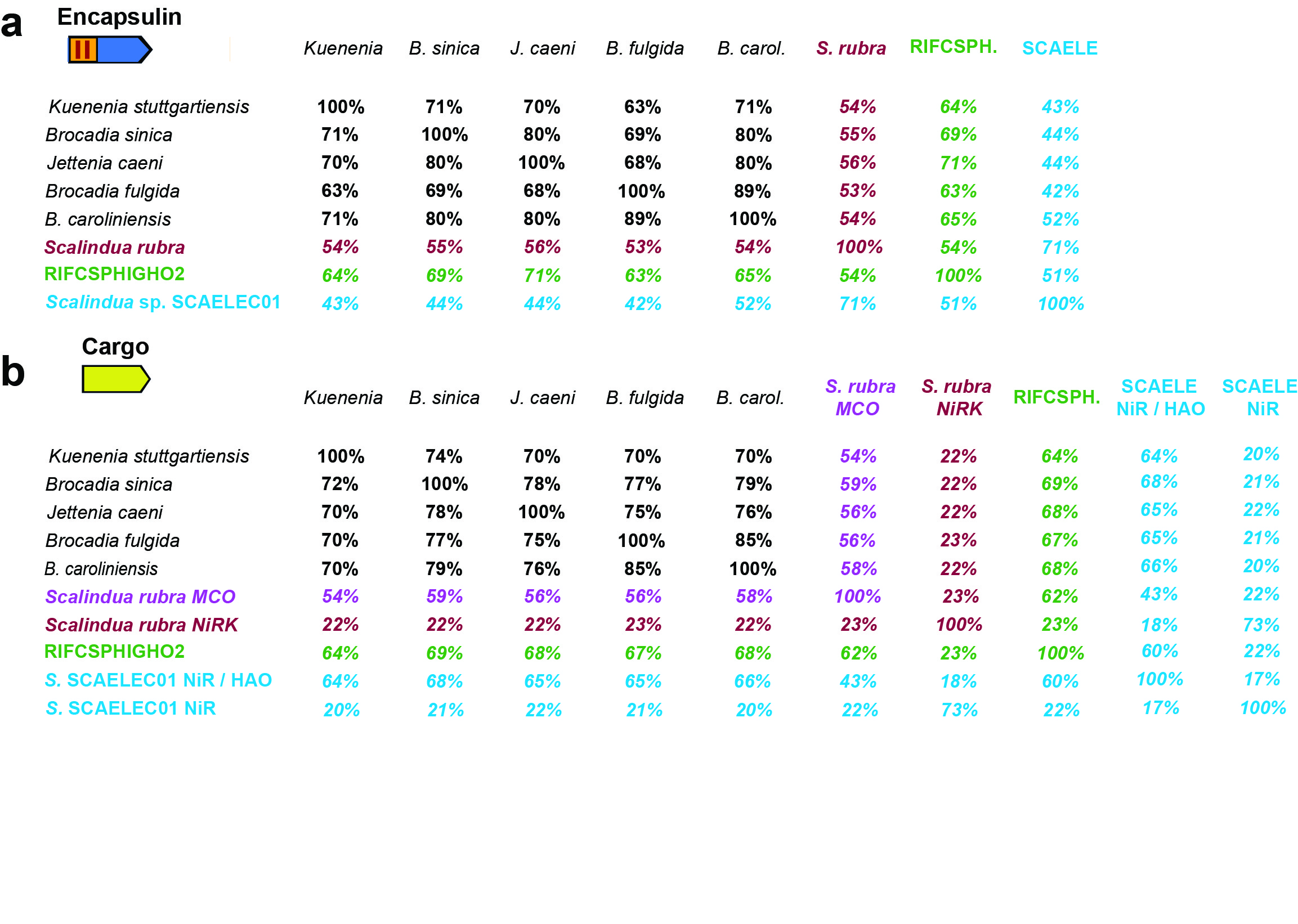
**

**Figure S7:** Percent identities at the amino acid level between all anammox and Planctomycete encapsulins and cargo proteins.
